# Xylanilyticolides A–C, Three New Compounds from Cultures of the Actinomycete *Promicromonospora xylanilytica* YIM 61515

**DOI:** 10.1007/s13659-018-0154-1

**Published:** 2018-02-13

**Authors:** Zhen-Xiong Wang, Shen Qin, Li-Hua Xu, He-Ping Chen, Huan Sun, Rong Huang, Zheng-Hui Li, Tao Feng, Ji-Kai Liu

**Affiliations:** 1 School of Pharmaceutical Sciences, South-Central University for Nationalities, Wuhan, 430074 China; 2 Yunnan Institute of Microbiology, Yunnan University, Kunming, 650091 China

**Keywords:** Actinomycete, *Promicromonospora xylanilytica*, Xylanilyticolides, Cytotoxicity

## Abstract

**Abstract:**

Three new lactones, xylanilyticolides A–C (**1**–**3**), were isolated from cultures of the actinomycete *Promicromonospora xylanilytica* YIM 61515. Their structures were elucidated by 1D and 2D NMR spectroscopic data in conjunction with HRESIMS analysis. Compound **1** exhibited potent cytotoxicities against five human cancer cell lines HL-60, A-549, SMMC-7721, MCF-7 and SW480 with the IC_50_ values of 3.9, 15.2, 11.2, 5.9, and 4.7 μM, respectively.

**Graphical Abstract:**

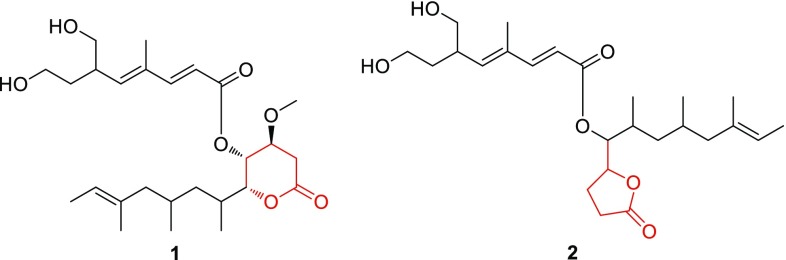

**Electronic supplementary material:**

The online version of this article (10.1007/s13659-018-0154-1) contains supplementary material, which is available to authorized users.

## Introduction

Actinomycetes are well-known for their ability to produce natural products with structural complexity and diverse biological activities [1, 2]. Salinomycin, isolated from the culture broth of an actinomycete strain of *Streptomyces*, shows remarkable toxicity for cancer stem cells [3, 4]. Actinomycetes, including some rare genera of actinomycetes [5], are also valuable sources in the discovery of natural antibiotics [6].

**Fig. 1:**
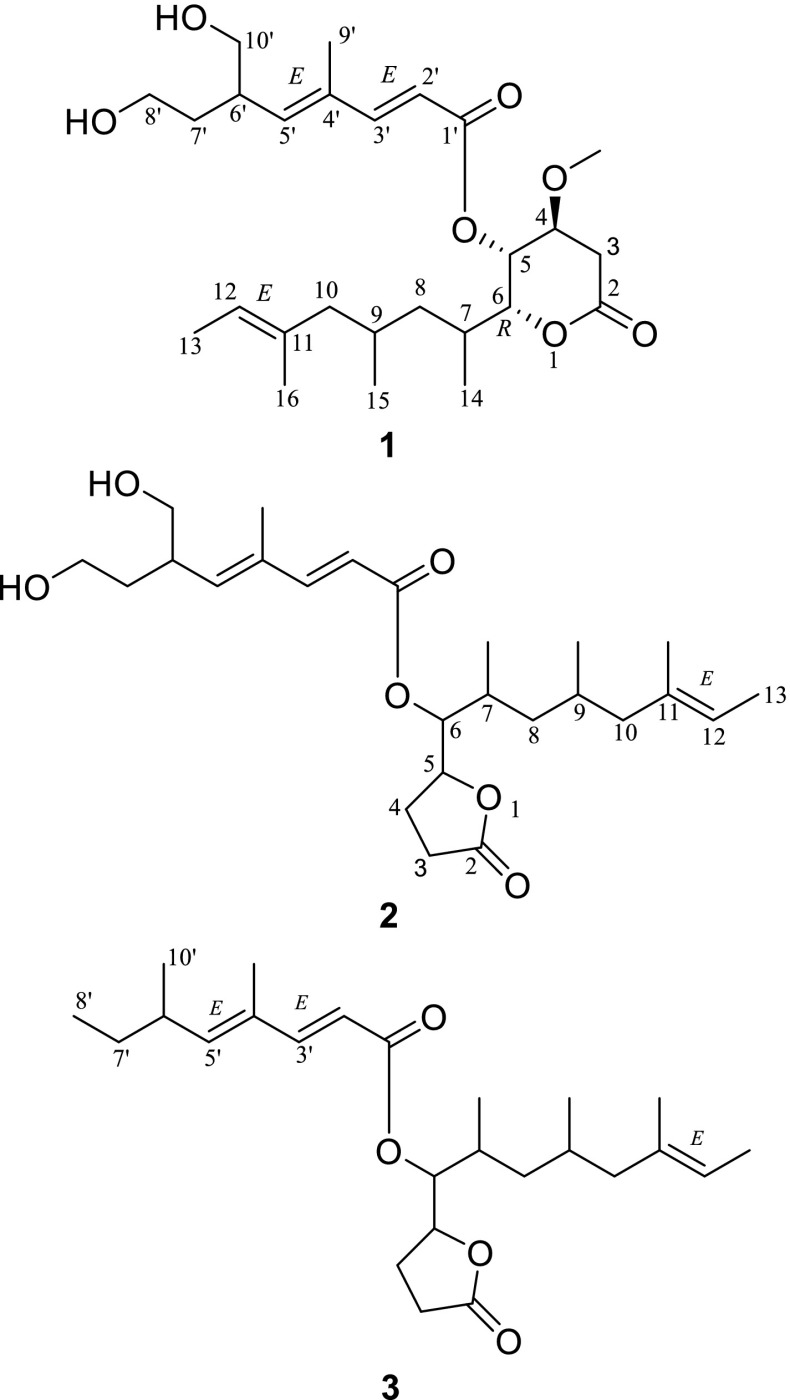
Structures of compounds **1**–**3**

Since the genus *Promicromonospora* was proposed by Krasil’nikov et al. in 1961 [7], there has been only one report of chemical investigation on this genus with discovery of a novel macrocyclic dilactone from *Promicromonospora* sp. RL26 in 2011 [8]. Herein, the investigation of secondary metabolites from the *Promicromonospora xylanilytica* YIM 61515 led to the discovery of three new products, xylanilyticolides A–C (**1**–**3**) (Fig. 1) [9]. Their structures were elucidated by analyses of 1D and 2D NMR spectroscopic methods. Their cytotoxicities against five human cancer cell lines were evaluated. Report herein are the isolation, structural elucidation and cytotoxicities of them.

## Results and Discussion

### Structural Elucidation of Xylanilyticolides A–C (**1**–**3**)

**Fig. 2:**
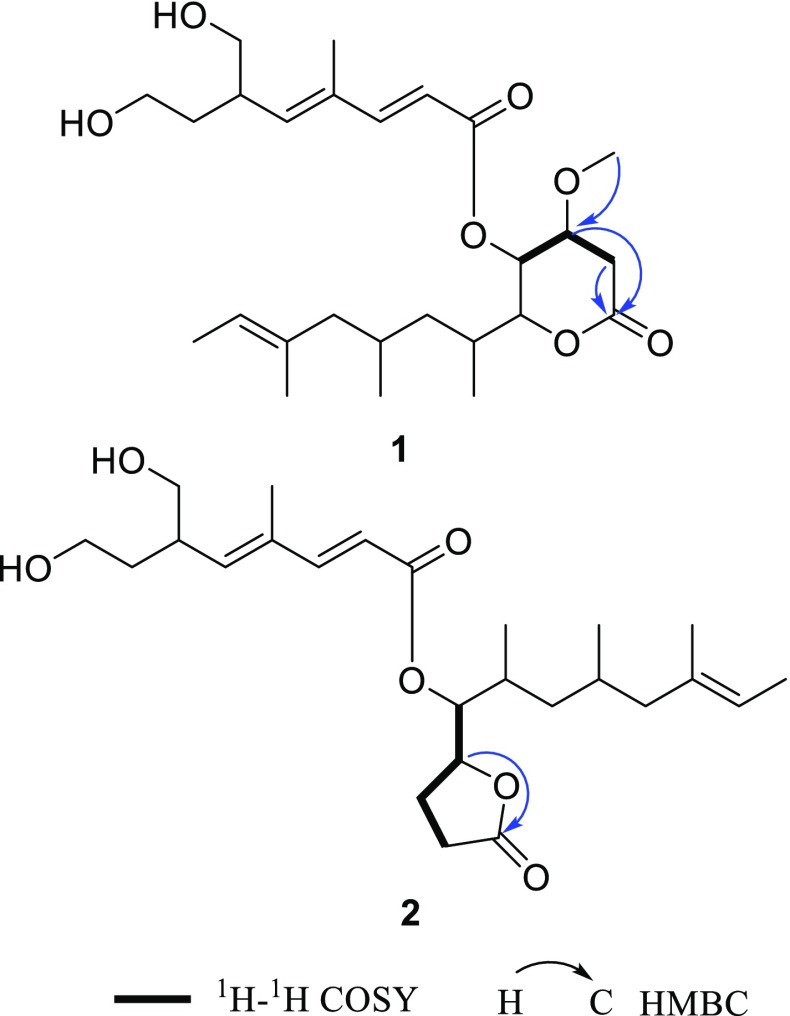
Key 2D NMR correlations of compounds **1** and **2**

Xylanilyticolide A (**1**), obtained as colorless oil, had a formula of C_26_H_42_O_7_ deduced by HRESIMS sodium adduct ion peak at *m/z* 489.2822 [M + Na]^+^ (calcd. for C_26_H_42_O_7_Na, 489.2823), requiring six degrees of unsaturation. The ^1^H NMR data displayed three methyl singlets at *δ*_H_ 1.84 (H-9′), 1.52 (H-16), 3.44 (OMe), three methyl doublets at *δ*_H_ 1.54 (H-13, d, *J* = 6.2 Hz), 1.09 (H-14, d, *J* = 6.6 Hz), 0.77 (H-15, d, *J* = 6.6 Hz), and two olefinic protons at *δ*_H_ 5.81 (H-2′, d, *J* = 15.7 Hz), 7.34 (H-3′, d, *J* = 15.7 Hz). The ^13^C NMR and DEPT spectra (Table 1) exhibited signals for 26 carbon atoms, including two carbonyls (*δ*_C_ 165.9, 169.5), six olefinic carbons (*δ*_C_ 115.3, 120.2, 134.4, 134.8, 143.7, 151.1), six methyls (*δ*_C_ 12.8, 13.5, 15.7, 16.1, 20.7, 57.3). The above data coupled with a literature survey indicated that compound **1** was analogous to the reported compound rasfonin, an apoptosis inducer in *Ras*-dependent cells isolated from the fungus *Talaromyces* sp. [10]. Comparing with the reported structure, the two distinct differences in compound **1** were the reduction of olefinic bond in C-3 and the substitution of a methoxy in C-4. The ^1^H-^1^H COSY correlations between H-3 (*δ*_H_ 2.79, 2.67)/H-4 (*δ*_H_ 3.70), H-4/H-5 (*δ*_H_ 5.29), and HMBC correlation from –OCH_3_ (*δ*_H_ 3.44) to C-4 (*δ*_C_ 74.1), from H-3 (*δ*_H_ 2.79, 2.67) and H-4 to C-2 (*δ*_C_ 169.5) supported these changes (Fig. 2).

**Table 1:** ^1^H (600 MHz) and ^13^C (150 MHz) NMR spectroscopic data of **1**–**3** (*δ* in ppm, *J* in Hz)

No.	**1** (CDCl_3_)		**2** (acetone-*d*_6_)		**3** (methanol-*d*_4_)	
	*δ*_C_, type	*δ*_H_	*δ*_C_, type	*δ*_H_	*δ*_C_, type	*δ*_H_
2	169.5, C		176.8, C		179.5, C	
3	32.8, CH_2_	2.79 (17.8, 5.5)	28.8, CH_2_	2.53, ddd (17.5, 9.5, 9.5)	29.3, CH_2_	2.58, ddd (17.5, 10.0, 8.9)
		2.67 (17.8, 3.2)		2.47, ddd (17.5, 9.5, 4.5)		2.49, overlapped
4	74.1, CH	3.70, ddd (5.5, 3.2, 3.2)	25.2, CH_2_	2.38, m; 1.98, m	25.5, CH_2_	2.35, m; 1.95, m
5	66.1, CH	5.29, dd (3.2, 1.5)	80.5, CH	4.79, ddd (7.4, 7.4, 6.5)	81.9, CH	4.81, ddd (7.4, 7.4, 6.4)
6	81.3, CH	4.31, dd (8.7, 1.5)	76.7, CH	5.06, dd (6.5, 4.0)	77.3, CH	5.04, dd (6.4, 4.0)
7	31.7, CH	1.99, m	31.9, CH	2.03, m	32.4, CH	2.01, overlapped
8	40, CH_2_	1.22, br. dd (13.3, 5.0)	41.7, CH_2_	1.36, ddd (14.4, 7.6, 6.3)	41.9, CH_2_	1.37, m
		1.01, ddd (13.3, 9.0, 3.8)		0.94, m		0.91, m
9	27.9, CH	1.67, m	28.3, CH	1.79, m	28.7, CH	1.75, m
10	46.4, CH_2_	2.00, overlapped	48.5, CH_2_	1.97, overlapped	29.6, CH_2_	1.92, overlapped
		1.44, dd (13.1, 9.8)		1.68, dd (13.0, 8.5)		1.72, m
11	134.4, C		135.2, C		135.5, C	
12	120.2, CH	5.12, q (6.2)	120.5, CH	5.15, q (6.6)	121.0, CH	5.15, q (6.6)
13	13.5, CH_3_	1.54, d (6.2)	13.4, CH_3_	1.52, d (6.6)	13.5, CH_3_	1.53, d (6.6)
14	16.1, CH_3_	1.09, d (6.6)	15.6, CH_3_	0.98, d (6.8)	15.7, CH_3_	0.99, d (6.8)
15	20.7, CH_3_	0.77, d (6.6)	20.2, CH_3_	0.82, d (6.5)	20.4, CH_3_	0.84, d (6.3)
16	15.7, CH_3_	1.52, s	15.6, CH_3_	1.54, s	15.7, CH_3_	1.54, s
1′	165.9, C		167.0, C		168.5, C	
2′	115.3, CH	5.81, d (15.7)	116.2, CH	5.87, d (15.7)	115.9, CH	5.85, d (15.6)
3′	151.1, CH	7.34, d (15.7)	150.9, CH	7.34, d (15.7)	150.3, CH	7.34, d (15.6)
4′	134.8, C		134.7, C		133.1, C	
5′	143.7, CH	5.78, d (10.0)	145.6, CH	5.84, d (10.0)	152.2, CH	5.73, d (10.0)
6′	39.4, CH	2.89, m	39.8, CH	2.89, m	36.2, CH	2.52, m
7′	34.9, CH_2_	1.80, m; 1.61, m	35.7, CH_2_	1.82, m; 1.48, m	31.1, CH_2_	1.46, m; 1.33, m
8′	60.8, CH_2_	3.74, m; 3.61, overlapped	60.4, CH_2_	3.59, m; 3.49, m	12.3, CH_3_	0.87, t (7.4)
9′	12.8, CH_3_	1.84, s	12.8, CH_3_	1.87, s	12.6, CH_3_	1.83, s
10′	66.0, CH_2_	3.63, overlapped	66.0, CH_2_	3.53, s, 2H	20.5, CH_3_	1.02, d (6.6)
		3.58, overlapped				
–OMe	57.3, CH_3_	3.44, s

**Fig. 3:**
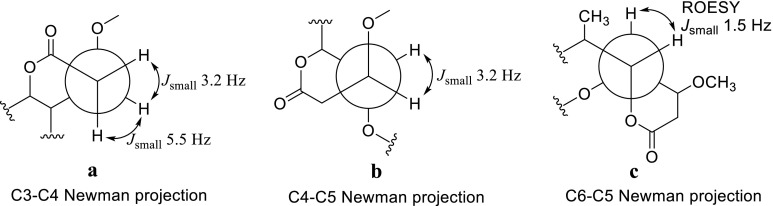
Newman projection and key ROESY correlations in compound **1**

Δ^2′^ was established as *E* configuration, which could be confirmed by the coupling constant (see Table 1). Verified by the ROESY correlation between H-3′ (*δ*_H_ 7.34)/H-5′ (*δ*_H_ 5.78), Δ^4′^ could identified as *E* configuration. Δ^11^ was *E* configuration, which could be identified by the H-12 (*δ*_H_ 5.12)/H-10 (*δ*_H_ 2.00, 1.44) ROESY correlation. From the Newman projection (Fig. 3), the *J*_H-3/H-4_ of 3.2 Hz and 5.5 Hz, *J*_H-4/H-5_ of 3.2 Hz, *J*_H-6/H-5_ of 1.5 Hz as well as H-5/H-6 ROESY correlation were observed, which could confirmed the relative configuration of the lactone ring as 4S*, 5R*, 6R*. However, the stereoconfigurations of C-7, C-9, and C-6′ can’t be determined by the ROESY experiment. The calculated ECD and single crystal X-ray diffraction were also in failure. Therefore, the structure of compound **1** was deduced as xylanilyticolide A.

Compound **2** was obtained as colorless oil. Its molecular formula was assigned as C_25_H_40_O_6_ by HRESIMS ion peak at *m/z* 459.2723 [M + Na]^+^ (calcd. for C_25_H_40_O_6_Na, 459.2717), implying the presence of six degrees of unsaturation. The ^1^H NMR spectrum of **2** illustrated the presence of two methyl singlets at *δ*_H_ 1.54 (H-16) and 1.87 (H-9′); two oxygenated methylenes at *δ*_H_ 3.59/3.49 (H-8′), 3.53 (H-10′); four olefinic protons at *δ*_H_ 5.87 (H-2′), 7.34 (H-3′), 5.84 (H-5′), 5.15 (H-12); and two oxygenated methines at *δ*_H_ 4.79 (H-5), 5.06 (H-6). Combined ^13^C NMR data (Table 1) and HSQC spectrum indicated the presence of five methyls, seven methylenes, five saturated methines, four olefinic methines, two fully substituted carbons (*δ*_C_ 135.2 and 134.7), and two ketone carbons (*δ*_C_ 176.8 and 167.0). The ^1^H and ^13^C chemical shifts of compound **2** (Table 1) were highly similar to those of **1**, except the *δ*-lactone moiety in **1** was replaced by a *γ*-lactone moiety in **2**, which could be verified by the HMBC correlation between H-5 (*δ*_H_ 4.79)/C-2 (*δ*_C_ 176.8) and ^1^H-^1^H COSY correlations of H-6 (*δ*_H_ 5.06)/H-5 (*δ*_H_ 4.79), H-5 (*δ*_H_ 4.79)/H-4 (*δ*_H_ 2.38, 1.98), H-4 (*δ*_H_ 2.38, 1.98)/H-3 (*δ*_H_ 2.53,2.47) of compound **2** (Fig. 2). The *E/Z* configurations of the double bonds in compound **2** were deduced as the same with those of compound **1** by ROESY correlations of H-9′ with H-6′ and H-10′. However, the stereochemistry of C-7, C-9, and C-6′ can’t be established currently. The structure of compound **2** was, therefore, identified as xylanilyticolide B.

The molecular of compound **3** was established by HRESIMS ion peak at *m/z* 427.2817 [M + Na]^+^ (calcd. for C_25_H_40_O_4_Na, 427.2819). Detailed analyses of ^1^H and ^13^C NMR data of compound **3** revealed that compound **3** had the same skeleton with that of compound **2**. The main difference was that C-8′ and C-10′ in compound **3** was two methyl carbons rather than two hydroxymethyl groups in **2**. Detailed analysis of 2D NMR data suggested that other parts of **3** were the same to those of **2**. Hence the structure of **3** was elucidated as xylanilyticolide C.

### Cytotoxicity Assays

Compounds **1**–**3** were evaluated for their cytotoxicity activity utilizing an MTS cytotoxicity assay in vitro (DDP was used as a positive control). Compound **1** showed potent inhibitory activity against HL-60, A-549, SMMC-7721, MCF-7 and SW480 cells lines with the IC_50_ values of 3.9, 15.2, 11.2, 5.9, 4.7 μM, respectively (Table 2), while compounds **2** and **3** showed no significant cytotoxicity activity under the concentration of 40 μM.

**Table 2:** Cytotoxicities of **1** against five human cancer cell lines (IC_50_ μM)

Compound	HL-60	A-549	SMMC-7721	MCF-7	SW480
**1**	3.9	15.2	11.2	5.9	4.7
DDP^a^	4.6	27.4	28.0	29.3	30.0

^a^Positive control

## Experimental

### General Experimental Procedures

Optical rotations were recorded on a JASCO P-1020 digital polari-meter (Horiba, Kyoto, Japan). UV/Vis spectra were obtained using a Shimadzu UV2401PC spectrometer (Shimadzu, Kyoto, Japan). 1D and 2D NMR spectra were measured on a Bruker Avance III 600 MHz spectrometers (Bruker Biospin GmbH, Karlsruhe, Germany). HRESIMS were recorded on an Agilent 6200 Q-TOF MS system (Agilent Technologies, Santa Clara, CA, USA). Silica gel (200–300 mesh, Qingdao Haiyang Chemical Co., Ltd., P. R. China) was used for chromatography column. Size exclusion chromatography was performed on Sephadex LH-20 (GE, Healthcare) columns. MPLC separation was conducted on a Biotage Isolera One using a Spherical C18 column. Semi-preparative HPLC was performed on an Agilent 1260 liquid chromatography system equipped with Zorbax SB-C18 columns (9.4 mm × 150 mm, particle size 5 μm).

### Actinomycete Materials and Culture Condition

The strain was identified as *P. xylanilytica* YIM 61515 by Prof. Shen Qin of Yunnan University. A voucher strain (HFC: 20160920-4A) was deposited at the School of Pharmaceutical Sciences, South-Central University for Nationalities, China.

The *P. xylanilytica* YIM 61515 strain was cultured in seed medium (glucose 20 g, peptone 2 g, yeast extract 2 g, soluble starch 5 g, K_2_HPO_4_ 0.5 g, MgSO_4_ 0.5 g, NaCl 4 g, CaCO_3_ 2 g in 1 L of deionized water, the pH was adjusted to 7.8 before autoclaving) at 25 °C with shaking at 150 rpm for 7 days. Then inoculated the seed culture into 50 Erlenmeyer flasks (500 mL), previously sterilized by autoclaving, each containing 100 g of rice and 100 mL of distilled water. All flasks were incubated at 25 °C for 70 days.

### Extraction and Isolation

Each flask added 200 mL of EtOAc, and the culture was homogenized. The suspension was extracted repeatedly with EtOAc (4 × 10 L), and the organic layer was evaporated to dryness under vacuum to yield a crude extract (49 g), which was fractionated by MPLC with a stepwise gradient of MeOH/H_2_O (v/v 10:90, 40:60, 70:30, 90:10, 100:0) to give nine fractions (Fr. A–J).

Fr. D (1.87 g) was subjected to 200–300 mesh silica gel column with stepwise elution petroleum ether–EtOAc (8:1–2:1) to obtain nine sub-fractions (D1–D9) based on TLC analyses. The third fraction Fr. D3 was purified by semi-preparative HPLC using a gradient elution (MeCN/H_2_O 50:50 2.5 mL/min) to give compound **1** (4.4 mg, colorless oil).

Fr. E (508 mg) was separated on Sephadex LH-20 (MeOH) to give four fractions (E1–E4), and compound **2** (3.9 mg) was obtained from Fr. E2 (66.9 mg) which purified with semi-preparative HPLC using an isocratic elution (MeCN/H_2_O 55:45, 3 mL/min).

Fr. F (1.9 g) was subjected to 200–300 mesh silica gel column eluting with petroleum ether–EtOAc 4:1 to give 5 fractions (F1–F5). Fr. F3 (98.4 mg) was subjected to semi-prep HPLC with isocratic elution (MeCN/H_2_O 90:10, 3 mL/min) to give compound **3** (0.8 mg).

- *Xylanilyticolide A* (**1**) colorless oil, [*α*]_D_^24^ 8.2 (*c* 0.11, MeOH); UV (MeOH) *λ*_max_(log *ε*) 202 (3.90), 269 (4.31) nm; ^1^H and ^13^C NMR data, see Table 1. HRESIMS *m/z* 489.2822 [M + Na]^+^ (calcd. for C_26_H_42_O_7_Na, 489.2823).
- *Xylanilyticolide B* (**2**) colorless oil, [*α*]_D_^24^ 1.7 (*c* 0.13, MeOH); UV (MeOH) *λ*_max_(log *ε*) 202 (3.85), 268 (4.31) nm; ^1^H and ^13^C NMR data, see Table 1. HRESIMS *m/z* 459.2723 [M + Na]^+^ (calcd. for C_25_H_40_O_6_Na, 459.2717).
- *Xylanilyticolide C* (**3**) colorless oil, [*α*]_D_^24^ 4.5 (*c* 0.07, MeOH); UV (MeOH) *λ*_max_(log *ε*) 202 (3.15), 262 (3.77), 544 (1.35) nm; ^1^H and ^13^C NMR data, see Table 1. HRESIMS *m/z* 427.2817 [M + Na]^+^ (calcd. for C_25_H_40_O_4_Na, 427.2819).

### Cytotoxicity

Five human cancer cell lines, breast cancer SK-BR-3, hepatocellular carcinoma SMMC-7721, human myeloid leukemia HL-60, pancreatic cancer PANC-1, and lung cancer A-549 were used. Cells were cultured in RPMI-1640 or in DMEM medium (Hyclone, Logan, UT, USA), supplemented with 10% fetal bovine serum (Hyclone, USA) in 5% CO_2_ at 37 °C. The cytotoxicity assay was performed according to 3-(4,5-dimethylthiazol-2-yl)-2,5-diphenyl tetrazolium bromide (MTT) method in 96-well microplates [11]. Briefly, 100 µL of adherent cells were seeded into each well of 96-well cell culture plates and allowed to adhere for 12 h before addition of test compounds, while suspended cells were seeded just before drug addition with initial density of 1 × 10^5^ cells/mL. Each tumor cell line was exposed to the test compound at concentrations of 0.0625, 0.32, 1.6, 8, and 40 *μ*M in triplicates for 48 h, and all tests were done in twice with cisplatin (Sigma, Los Angeles, USA) as a positive control. After compound treatment, cell viability was detected and a cell growth curve was graphed. IC_50_ values were calculated by Reed and Muench’s method [12].

## Electronic supplementary material

Below is the link to the electronic supplementary material.

Supplementary material 1 (PDF 3875 kb)
