# Supplementary material for: Xylanilyticolides A–C, Three New Compounds from Cultures of the Actinomycete Promicromonospora xylanilytica YIM 61515
Source: Nat Prod Bioprospect. 2018 Feb 13;8(2):91–5. doi: 10.1007/s13659-018-0154-1 (PMC5913047; doi:10.1007/s13659-018-0154-1)
Supplement: Supplementary file 1 — Supplementary material 1 (PDF 3875 kb) [file 13659_2018_154_MOESM1_ESM.pdf]

Supporting information for

Xylanilyticolides A–C, three New Compounds from Cultures of the Actinomycete *Promicromonospora xylanilytica* YIM 61515

Zhen-Xiong Wang[1], Shen Qin[2], Li-Hua Xu[2], He-Ping Chen[1], Huan Sun[1], Rong Huang[1], Zheng-Hui Li[1], Tao Feng[1]*, Ji-Kai Liu[1]*

[1]School of Pharmaceutical Sciences, South-Central University for Nationalities, Wuhan 430074, China

[2]Yunnan Institute of Microbiology, Yunnan University, Kunming 650091, China

*Corresponding authors. E-mail addresses: tfeng@mail.scuec.edu.cn (T. Feng); jkliu@mail.kib.ac.cn (J.-K. Liu).

Figures	Content
S1	^{1}H NMR spectrum of compound **1** ($CDCl_3$).
S2	^{13}C NMR and DEPT spectra of compound **1** ($CDCl_3$).
S3	HSQC spectrum of compound **1** ($CDCl_3$).
S4	^{1}H–^{1}H COSY spectrum of compound **1** ($CDCl_3$).
S5	HMBC spectrum of compound **1** ($CDCl_3$).
S6	ROESY spectrum of compound **1** ($CDCl_3$).
S7	HRESIMS spectrum of compound **1**.
S8	UV spectrum of compound **1**.
S9	OR spectrum of compound **1**.
S10	^{1}H NMR spectrum of compound **2** (CD_3COCD_3).
S11	^{13}C NMR and DEPT spectra of compound **2** (CD_3COCD_3).
S12	HSQC spectrum of compound **2** (CD_3COCD_3).
S13	^{1}H–^{1}H COSY spectrum of compound **2** (CD_3COCD_3).
S14	HMBC spectrum of compound **2** (CD_3COCD_3).
S15	ROESY spectrum of compound **2** (CD_3COCD_3).
S16	HRESIMS spectrum of compound **2**.
S17	UV spectrum of compound **2**.
S18	OR spectrum of compound **2**.
S19	^{1}H NMR spectrum of compound **3** (CD_3OD).
S20	^{13}C NMR and DEPT spectra of compound **3** (CD_3OD).
S21	ROESY spectrum of compound **3** (CD_3OD).
S22	HRESIMS spectrum of compound **3**.
S23	UV spectrum of compound **3**.
S24	OR spectrum of compound **3**.

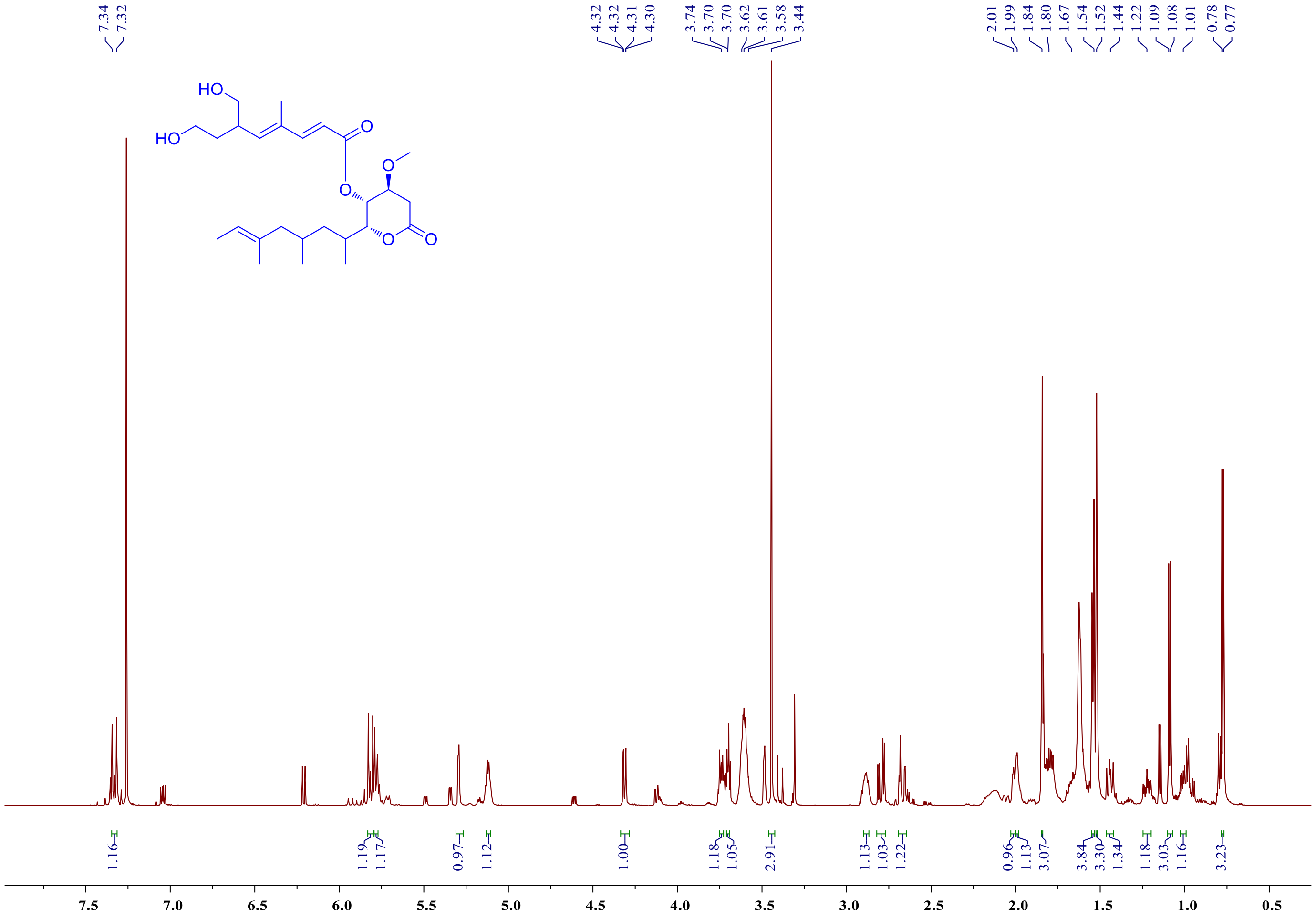

Figure S1. ^1H NMR spectrum of compound **1** ($CDCl_3$).

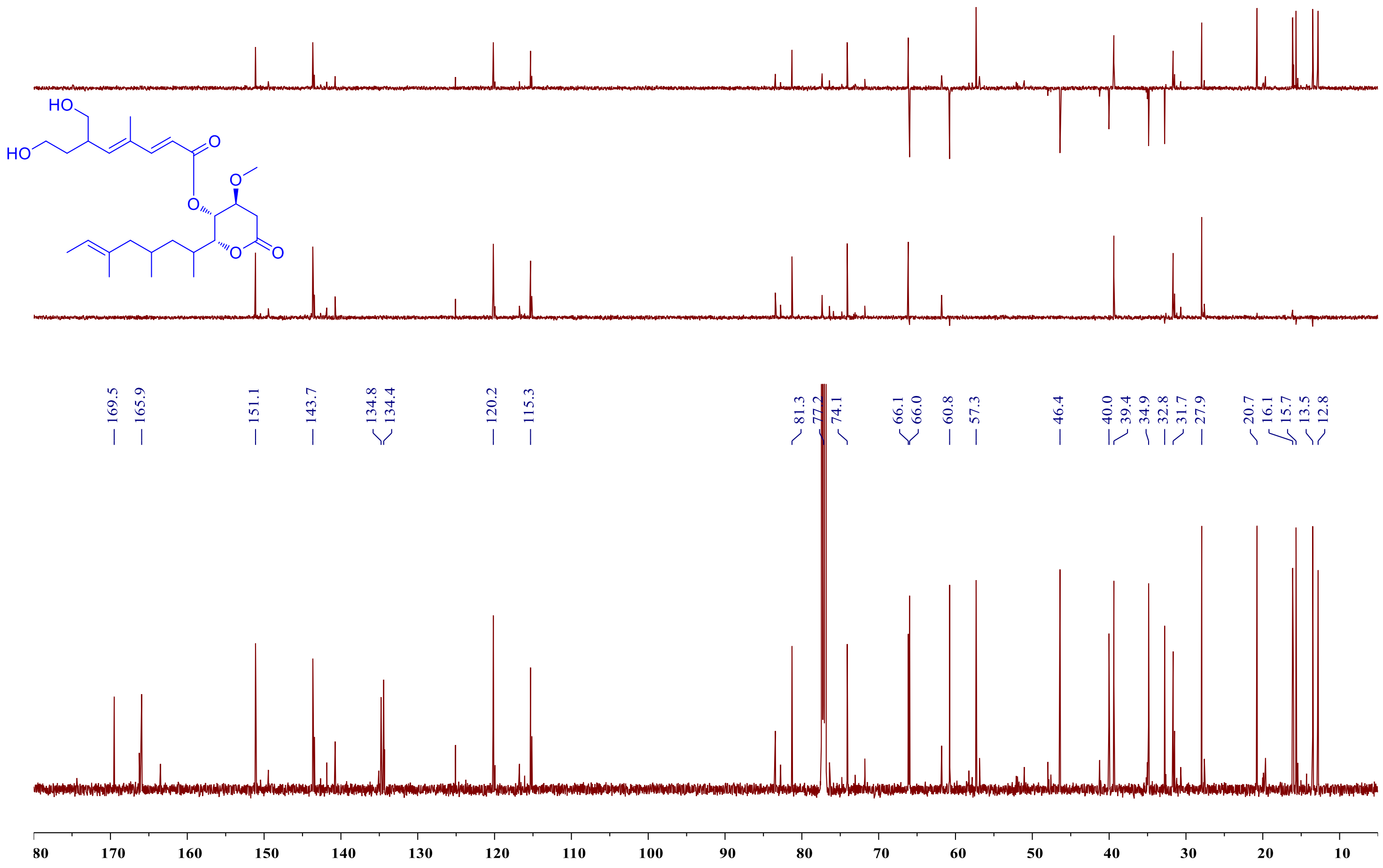

Figure S2. ^{13}C NMR and DEPT spectra of compound **1** ($CDCl_3$).

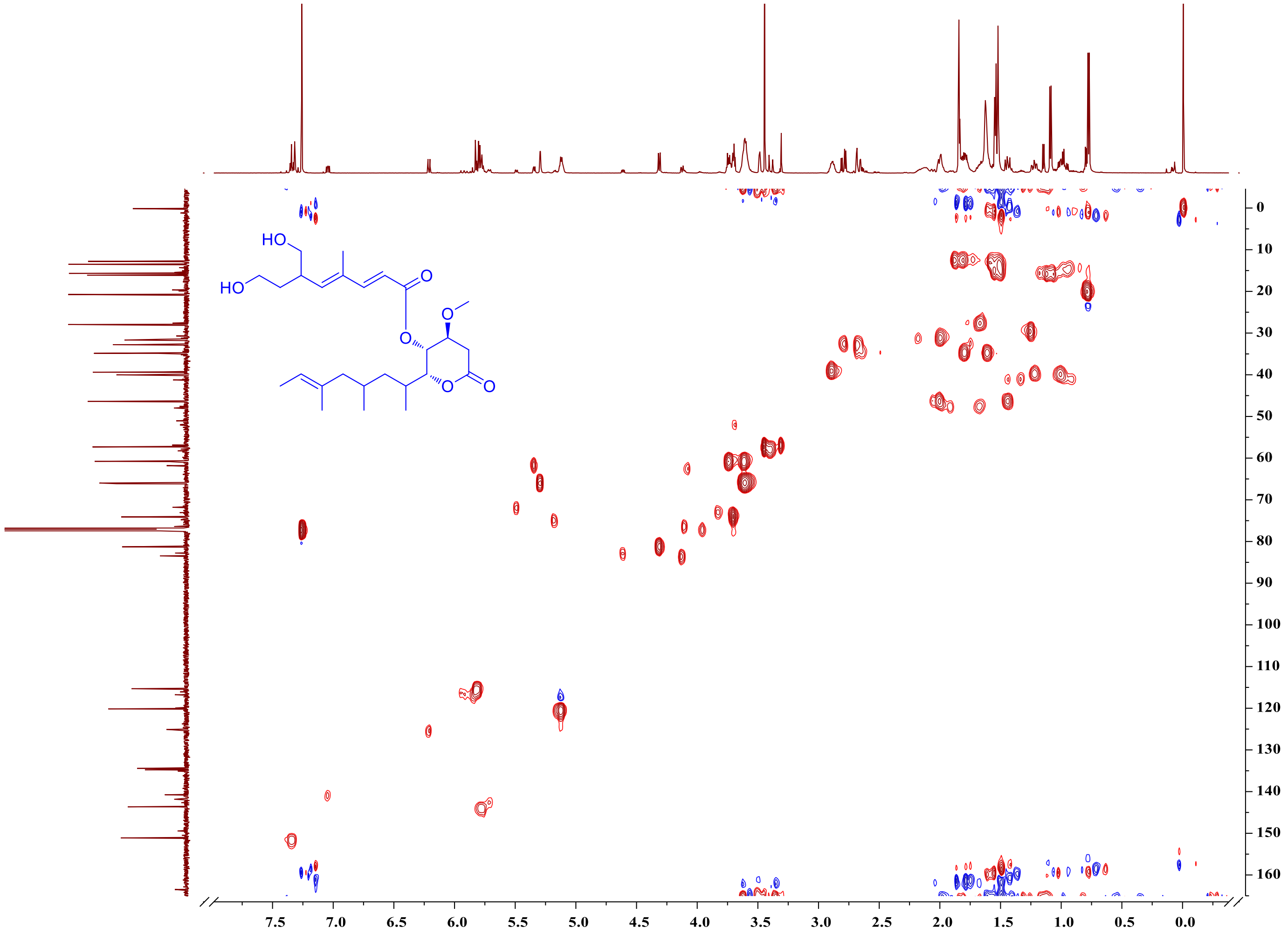

Figure S3. HSQC spectrum of compound **1** ($CDCl_3$).

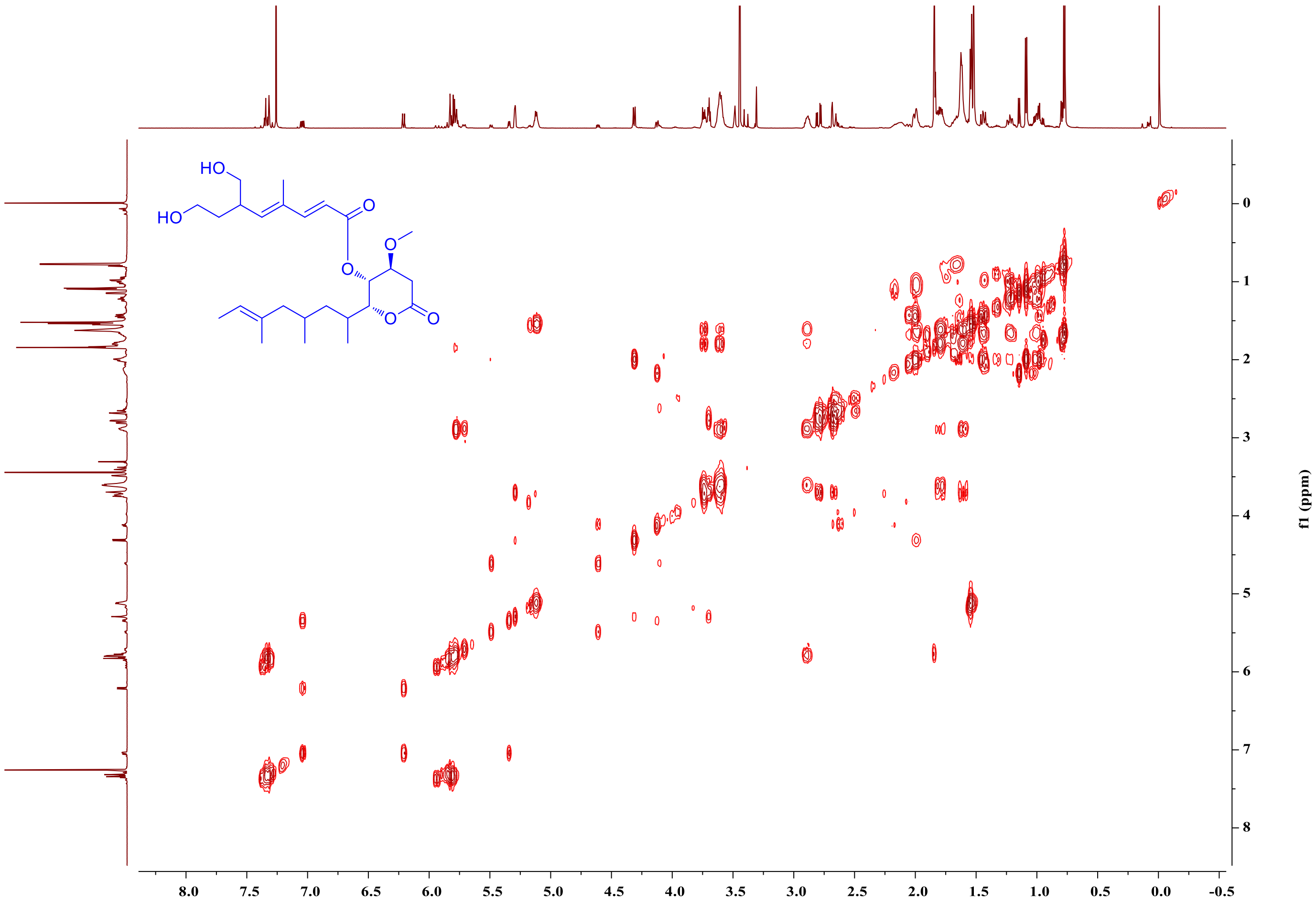

Figure S4. ^{1}H–^{1}H COSY spectrum of compound **1** ($CDCl_3$).

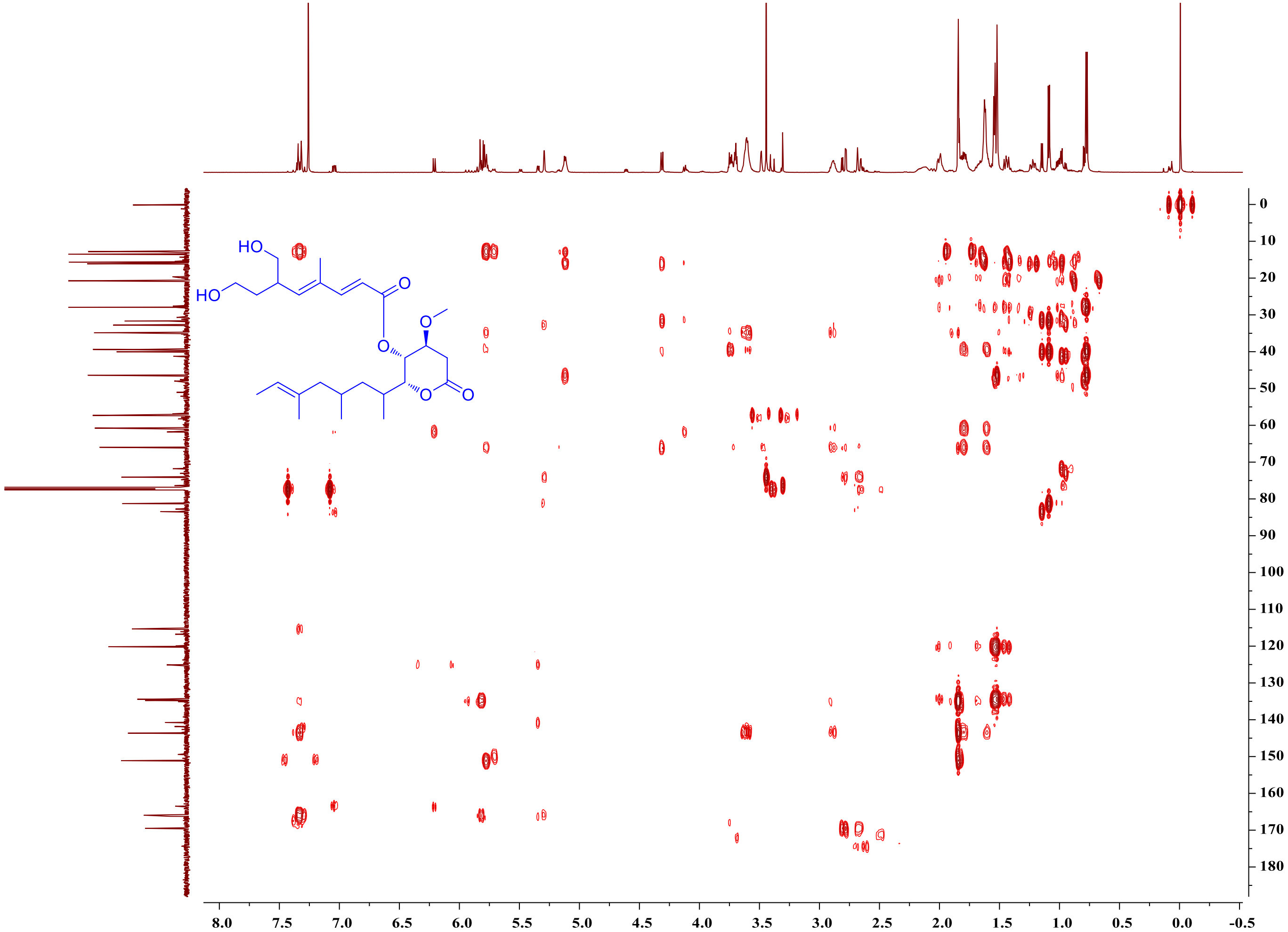

Figure S5. HMBC spectrum of compound **1** ($CDCl_3$).

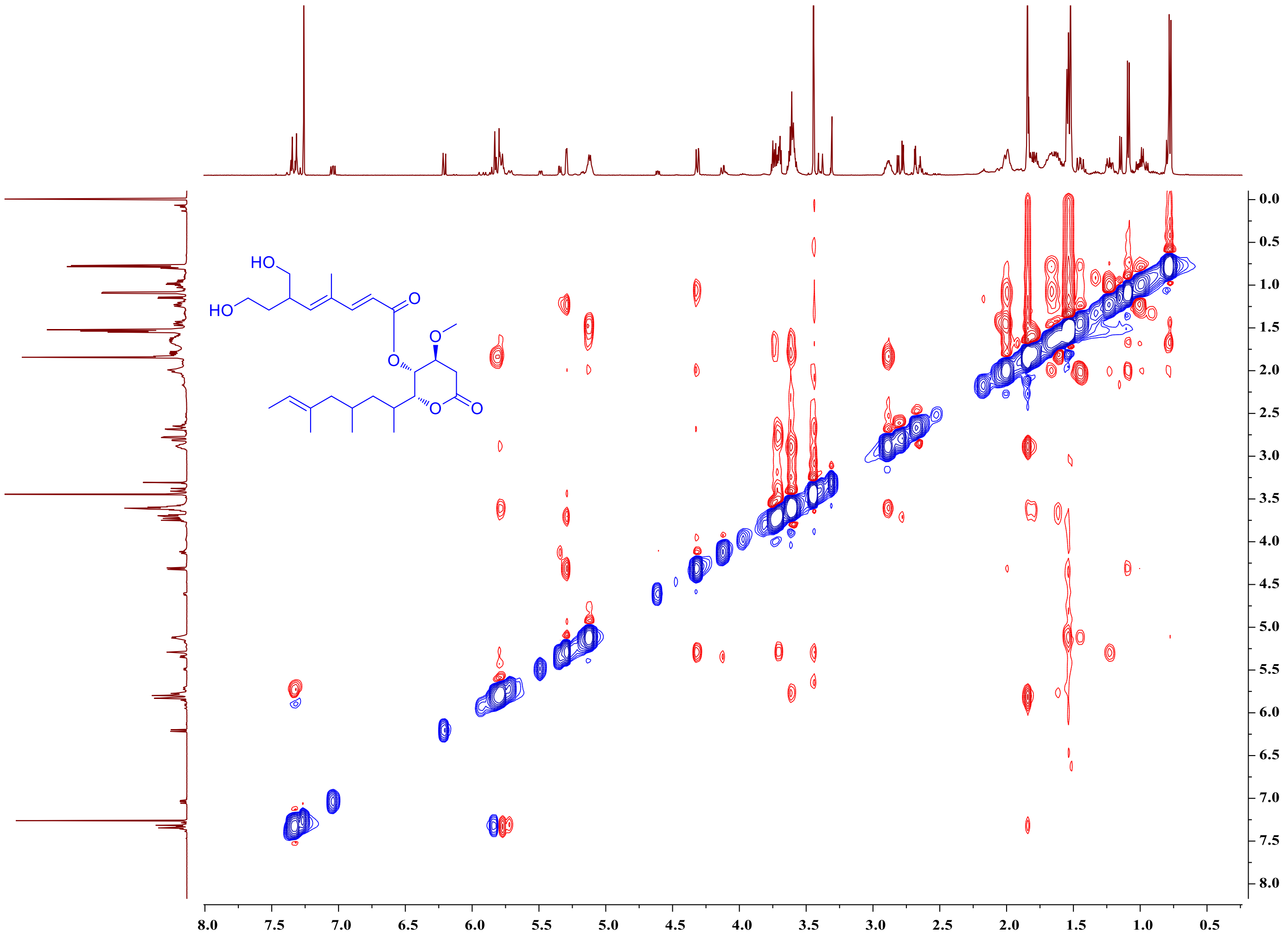

Figure S6. ROESY spectrum of compound **1** ($CDCl_3$).

Qualitative Analysis Report

**Data Filename**	lwz-42c.d	**Sample Name**	lwz-42c
**Sample Type**	Sample	**Position**	P1-D4
**Instrument Name**	Instrument 1	**User Name**	
**Acq Method**	SIBU.m	**Acquired Time**	7/5/2017 9:32:42 AM
**IRM Calibration Status**	Success	**DA Method**	Default.m
**Comment**			

Sample Group **Info.**
Acquisition SW 6200 series TOF/6500 series
Version Q-TOF B.05.01 (B5125.2)

User Spectra

Fragmentor Voltage	Collision Energy	Ionization Mode
135	0	ESI

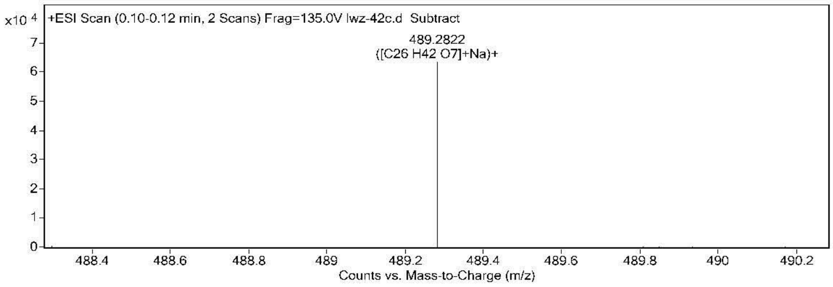

Peak List

m/z	z	Abund	Formula	Ion
489.2822	1	63364.65	C26 H42 O7	(M+Na)+
490.286	1	15862.02	C26 H42 O7	(M+Na)+
505.2564	1	74717.87		
506.2598	1	19312.89		
512.3571	1	20503.49		
521.3072	1	26736.8		
537.2806	1	21567.4		
540.3882	1	12954.47		
955.5738	1	23307.57		
987.5986	1	21044.09		

Formula Calculator Element Limits

Element	Min	Max
C	3	60
H	0	120
O	0	30
N	0	5

Formula Calculator Results

Formula	CalculatedMass	CalculatedMz	Mz	Diff. (mDa)	Diff. (ppm)	DBE
C26 H42 O7	466.2931	489.2823	489.2822	0.1	0.2	6.0000

--- End Of Report ---

Figure S7. HRESIMS spectrum of compound **1**.

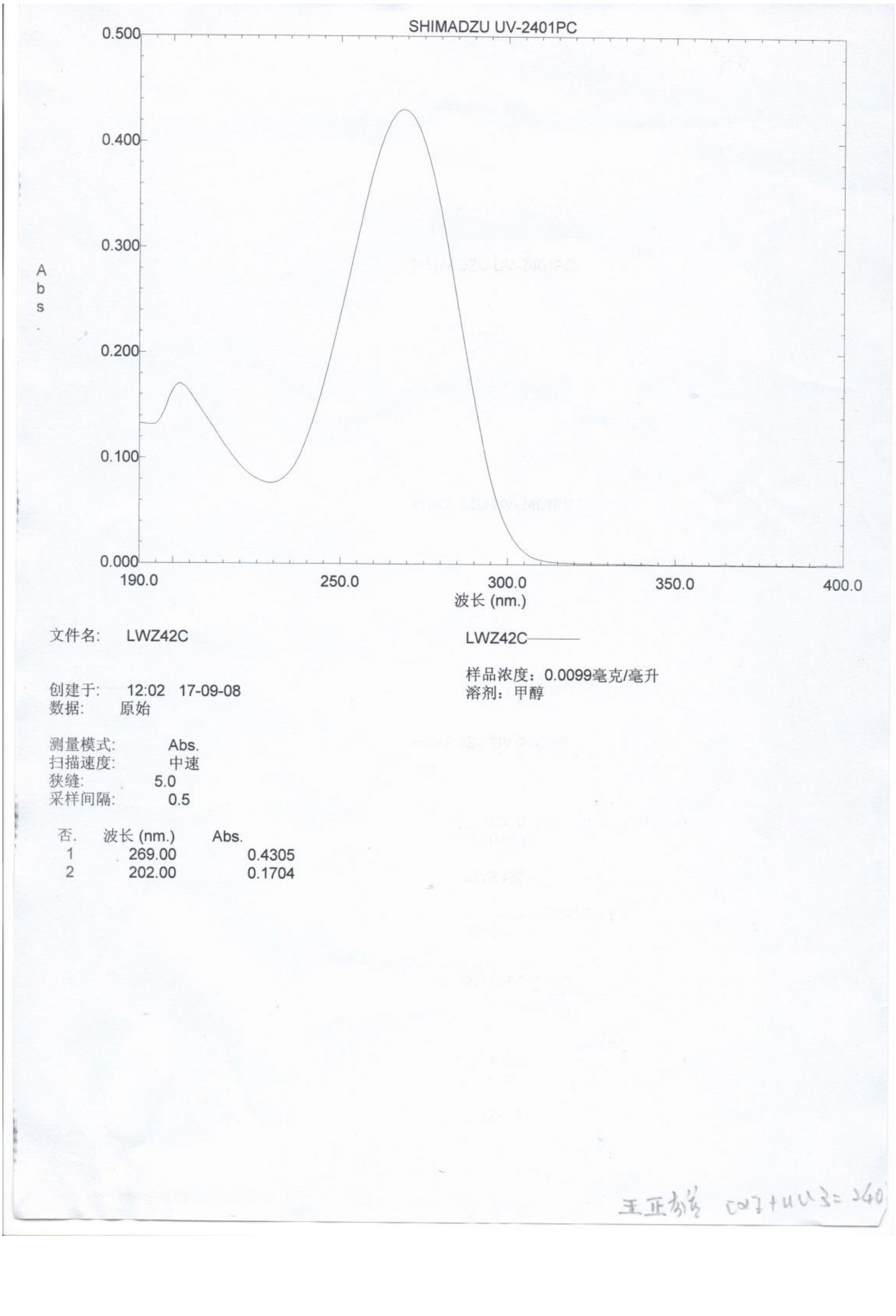

Figure S8. UV spectrum of compound **1**.

Optical rotation measurement

Model : P-1020 (A060460638)

No.	Sample	Mode	Data	Monitor<br>Blank	Temp.<br>Cell<br>Temp Point	Date<br>Comment<br>Sample Name	Light<br>Filter<br>Operator	Cycle Time<br>Integ Time	
No.1	35 (1/3)	Sp.Rot	-6.1820	-0.0034<br>0.0000	23.9<br>50.00<br>Cell	Wed Sep 06 20:44:33 2017<br>0.00110g/mL MeOH<br>LWZ42C	Na<br>589nm	2 sec<br>2 sec	
No.2	35 (2/3)	Sp.Rot	-8.9090	-0.0049<br>0.0000	23.9<br>50.00<br>Cell	Wed Sep 06 20:44:38 2017<br>0.00110g/mL MeOH<br>LWZ42C	Na<br>589nm	2 sec<br>2 sec	-8.2424°
No.3	35 (3/3)	Sp.Rot	-9.6360	-0.0053<br>0.0000	23.9<br>50.00<br>Cell	Wed Sep 06 20:44:44 2017<br>0.00110g/mL MeOH<br>LWZ42C	Na<br>589nm	2 sec<br>2 sec	

Figure S9. OR spectrum of compound **1**.

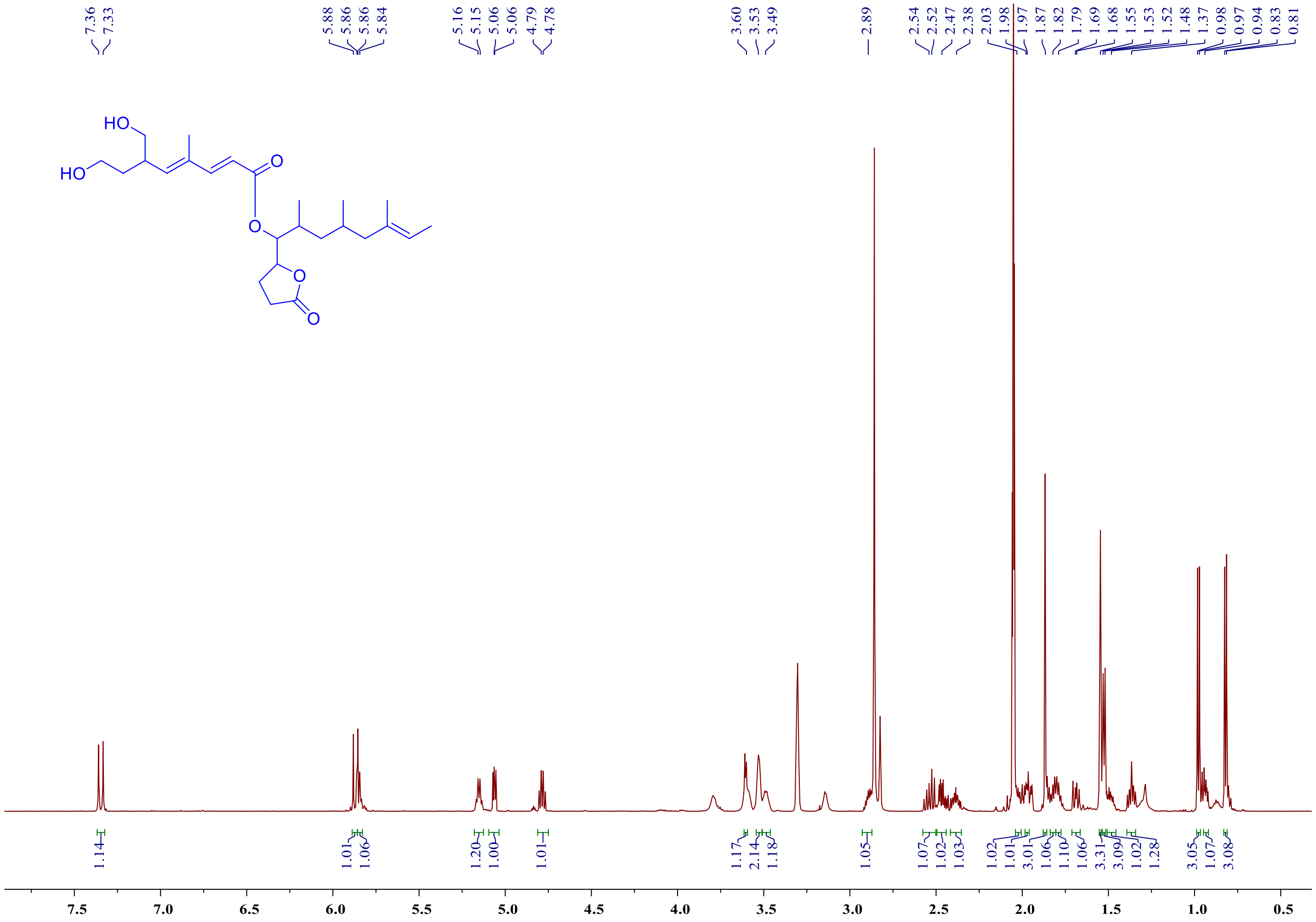

Figure S10. ^{1}H NMR spectrum of compound **2** (CD_3COCD_3).

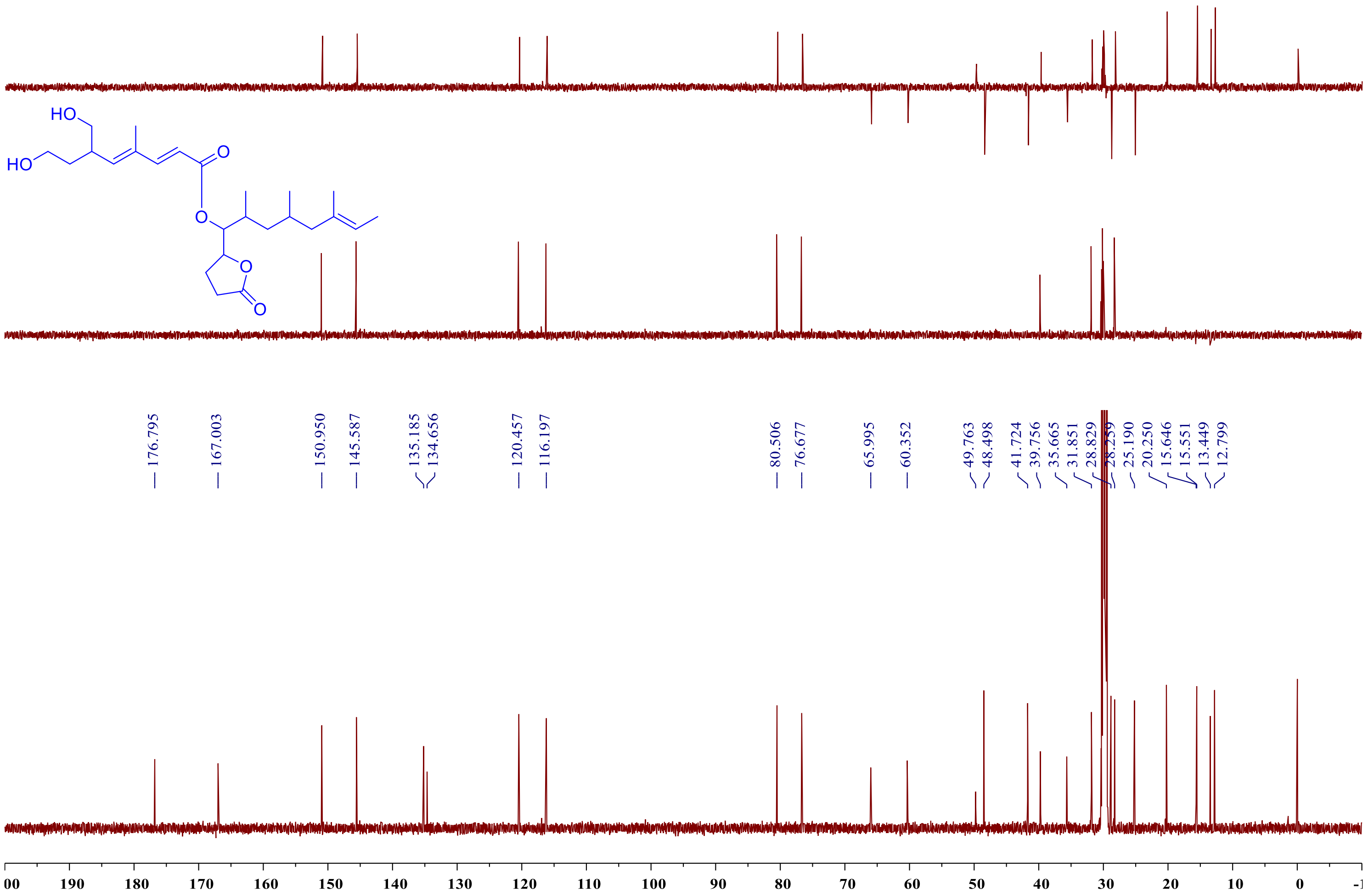

Figure S11. ^{13}C NMR and DEPT spectra of compound **2** (CD_3COCD_3).

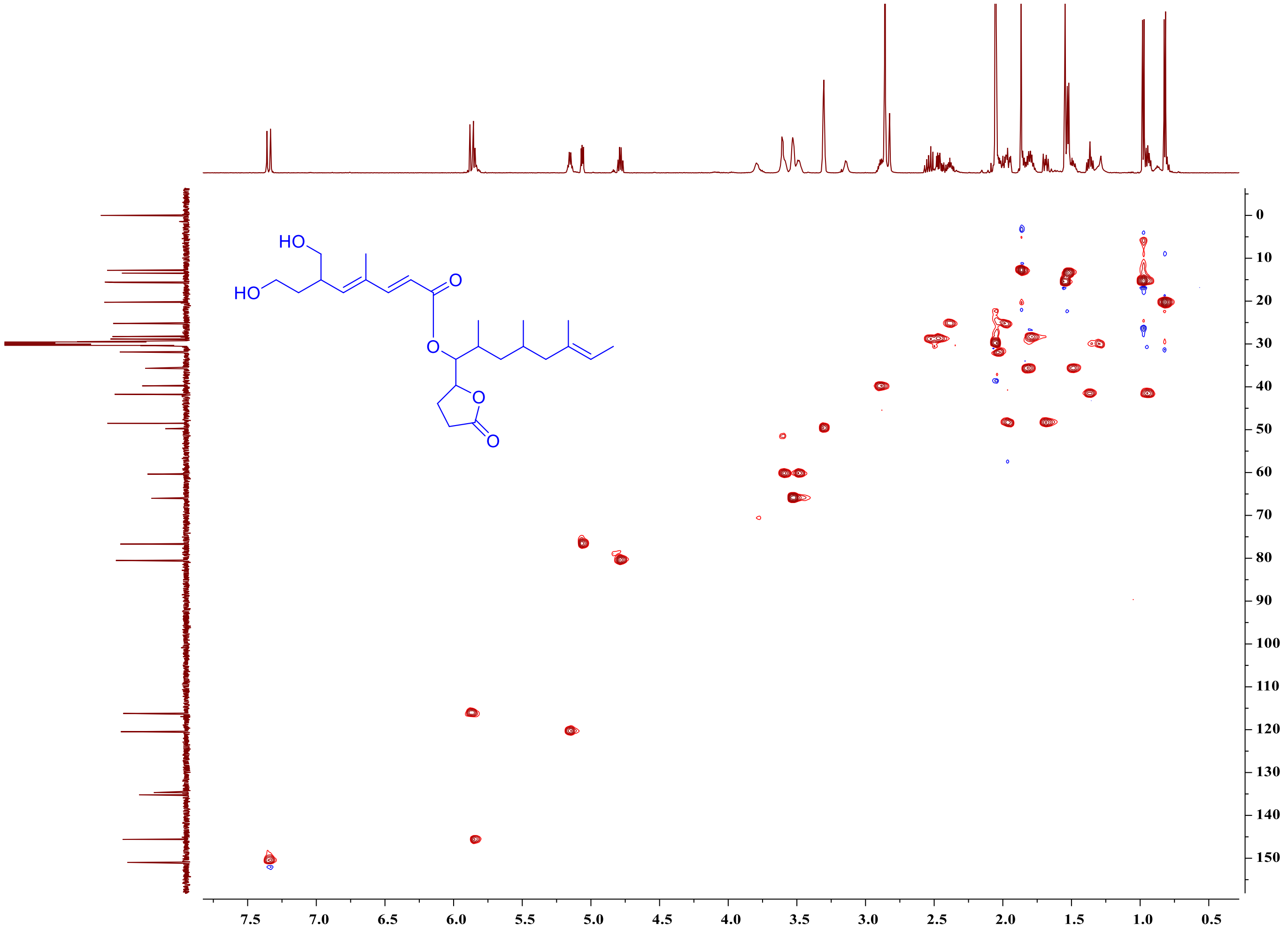

Figure S12. HSQC spectrum of compound **2** (CD_3COCD_3).

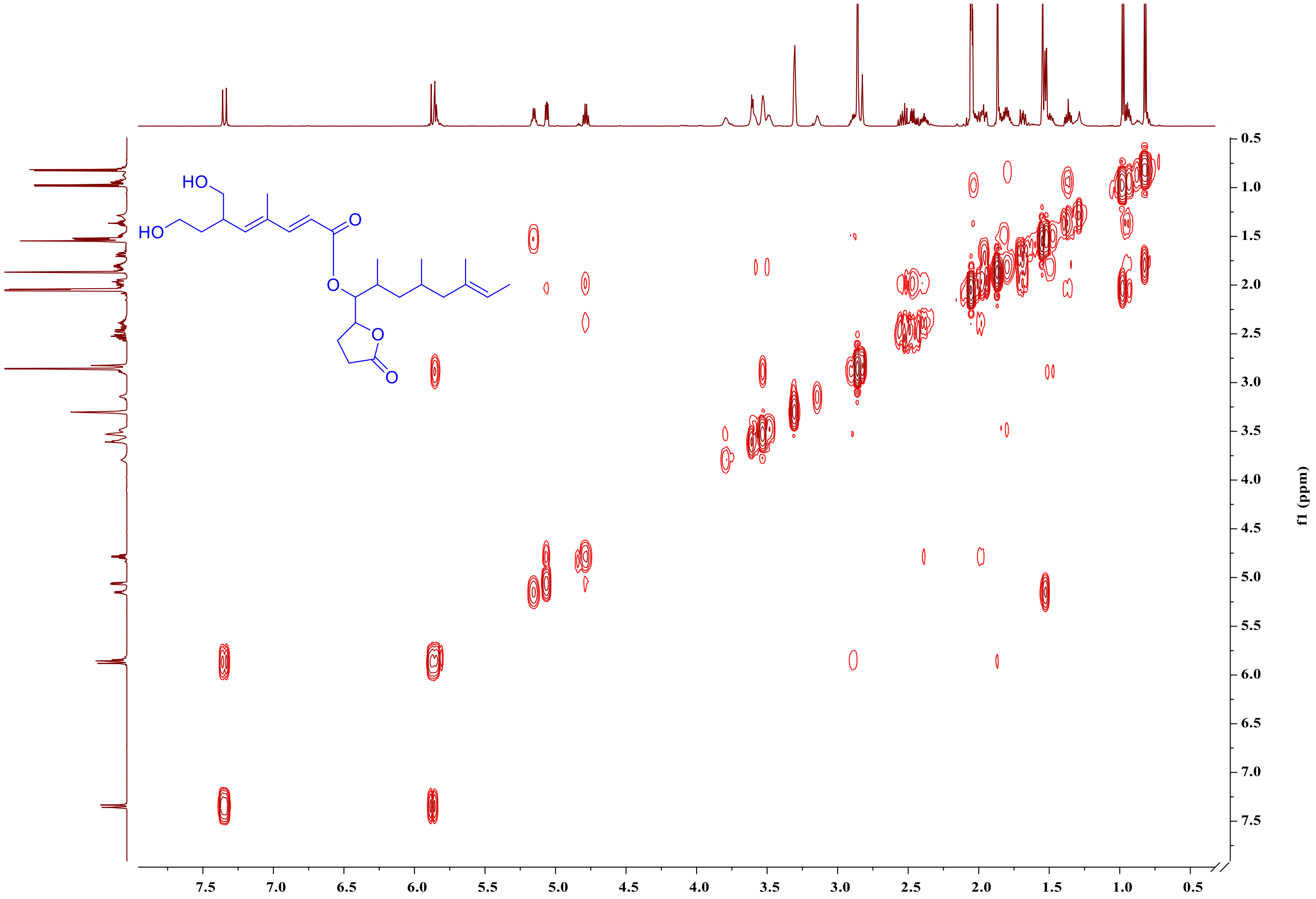

Figure S13. ^{1}H–^{1}H COSY spectrum of compound **2** (CD_3COCD_3).

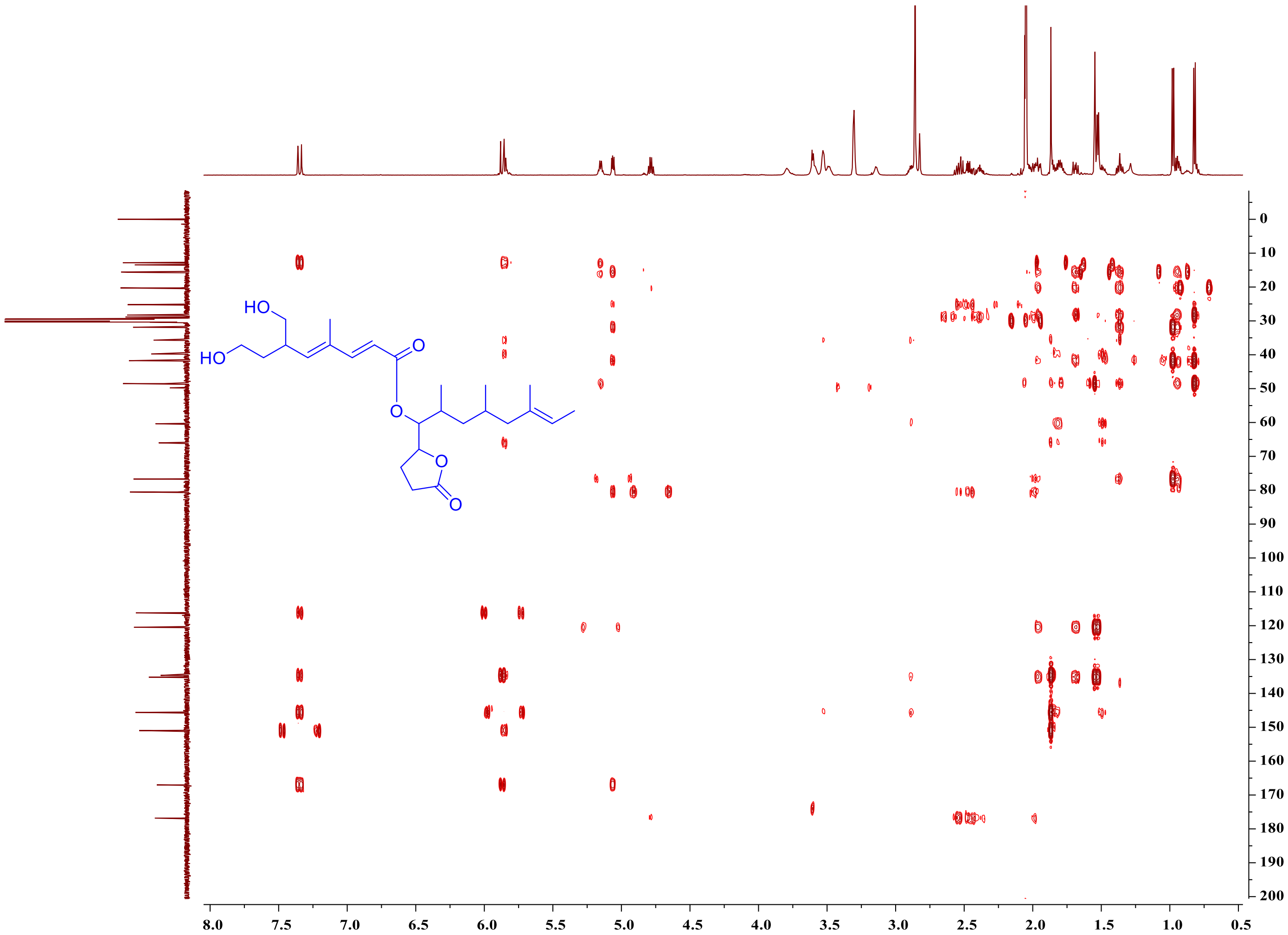

Figure S14. HMBC spectrum of compound **2** (CD_3COCD_3).

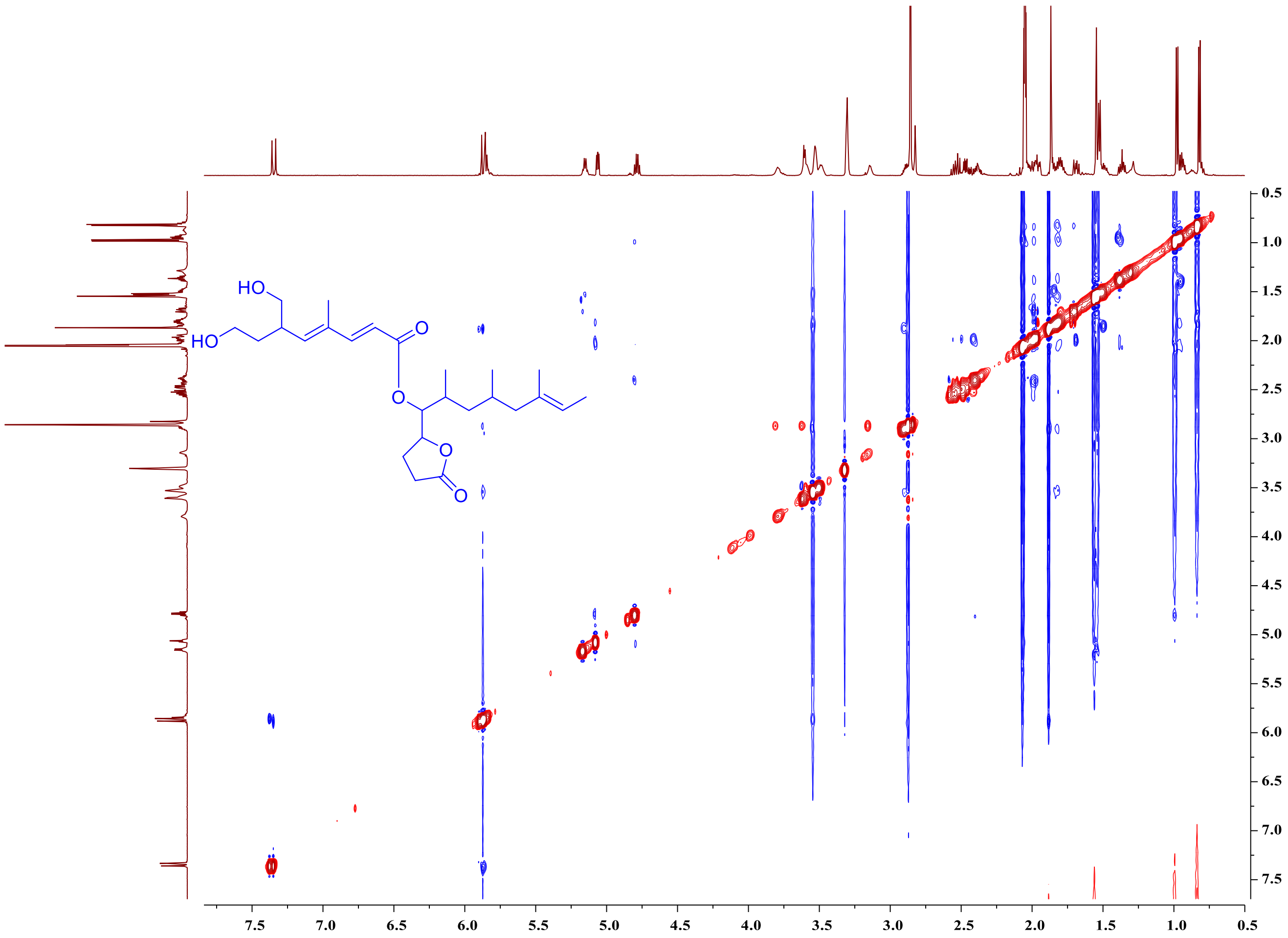

Figure S15. ROESY spectrum of compound **2** (CD_3COCD_3).

Qualitative Analysis Report

**Data Filename**	lwz-40b.d	**Sample Name**	lwz-40b
**Sample Type**	Sample	**Position**	P1-D2
**Instrument Name**	Instrument 1	**User Name**	
**Acq Method**	SIBU.m	**Acquired Time**	7/5/2017 9:30:19 AM
**IRM Calibration Status**	Success	**DA Method**	Default.m
**Comment**			

Sample Group **Info.**
Acquisition SW 6200 series TOF/6500 series
Version Q-TOF B.05.01 (B5125.2)

User Spectra

Fragmentor Voltage	Collision Energy	Ionization Mode
135	0	ESI

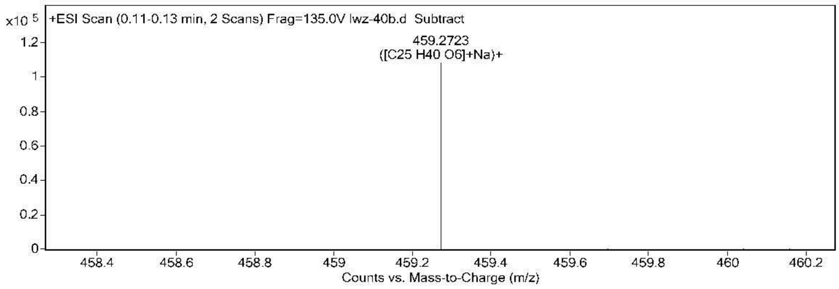

Peak List

m/z	z	Abund	Formula	Ion
454.3166	1	31739.01		
459.2723	1	108174.98	C25 H40 O6	(M+Na)+
475.2462	1	104542.75		
482.348	1	38916.8		
491.2973	1	28779.95		
895.554	1	70688.02		
896.5577	1	37002.55		
911.5278	1	79897.62		
912.5311	1	38656.14		
927.5799	1	51618.46		

Formula Calculator Element Limits

Element	Min	Max
C	3	60
H	0	120
O	0	30
N	0	5

Formula Calculator Results

Formula	CalculatedMass	CalculatedMz	Mz	Diff. (mDa)	Diff. (ppm)	DBE
C25 H40 O6	436.2825	459.2717	459.2723	-0.4	-1.0	6.0000

--- End Of Report ---

Figure S16. HRESIMS spectrum of compound **2**.

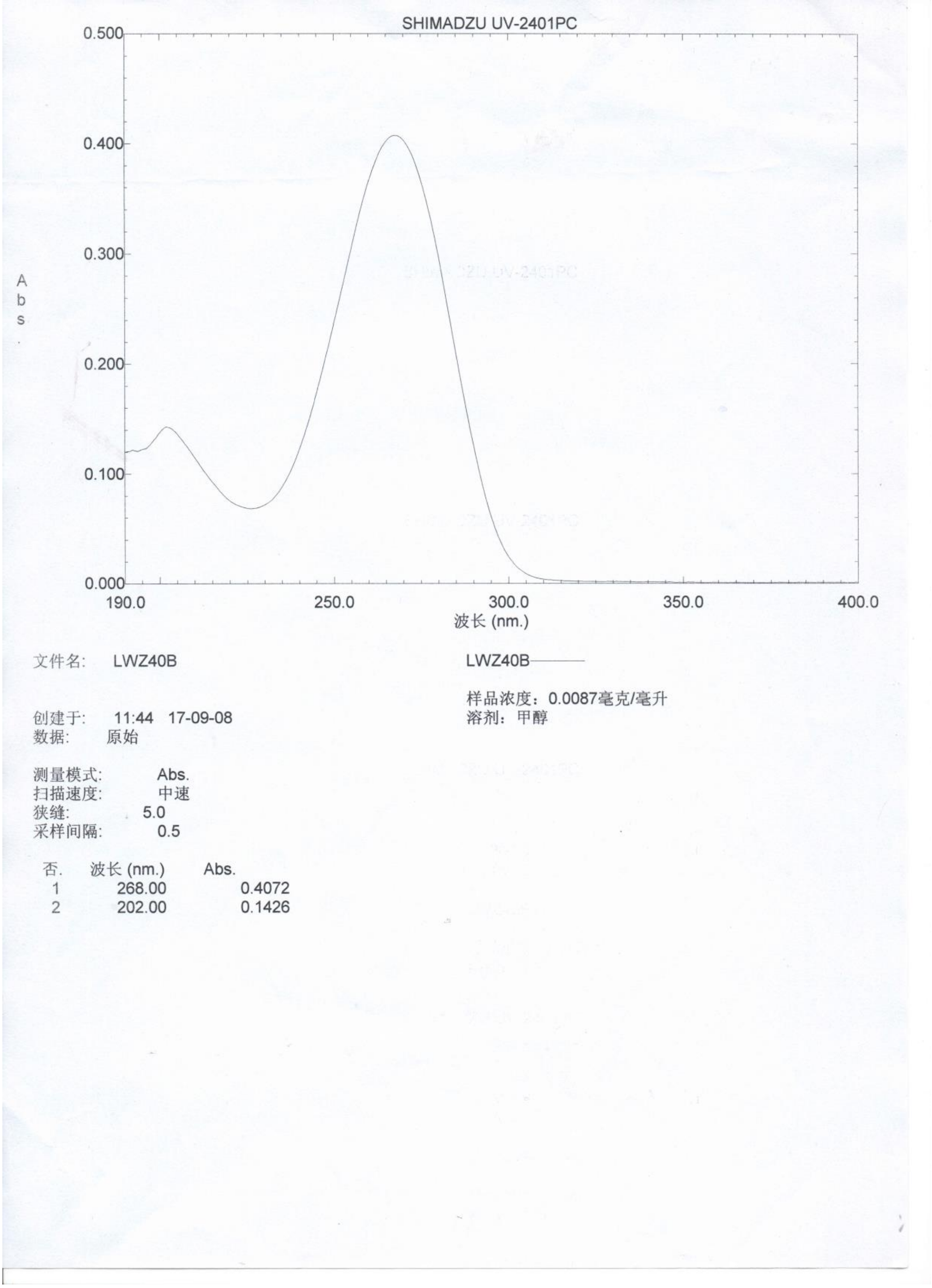

Figure S17. UV spectrum of compound **2**.

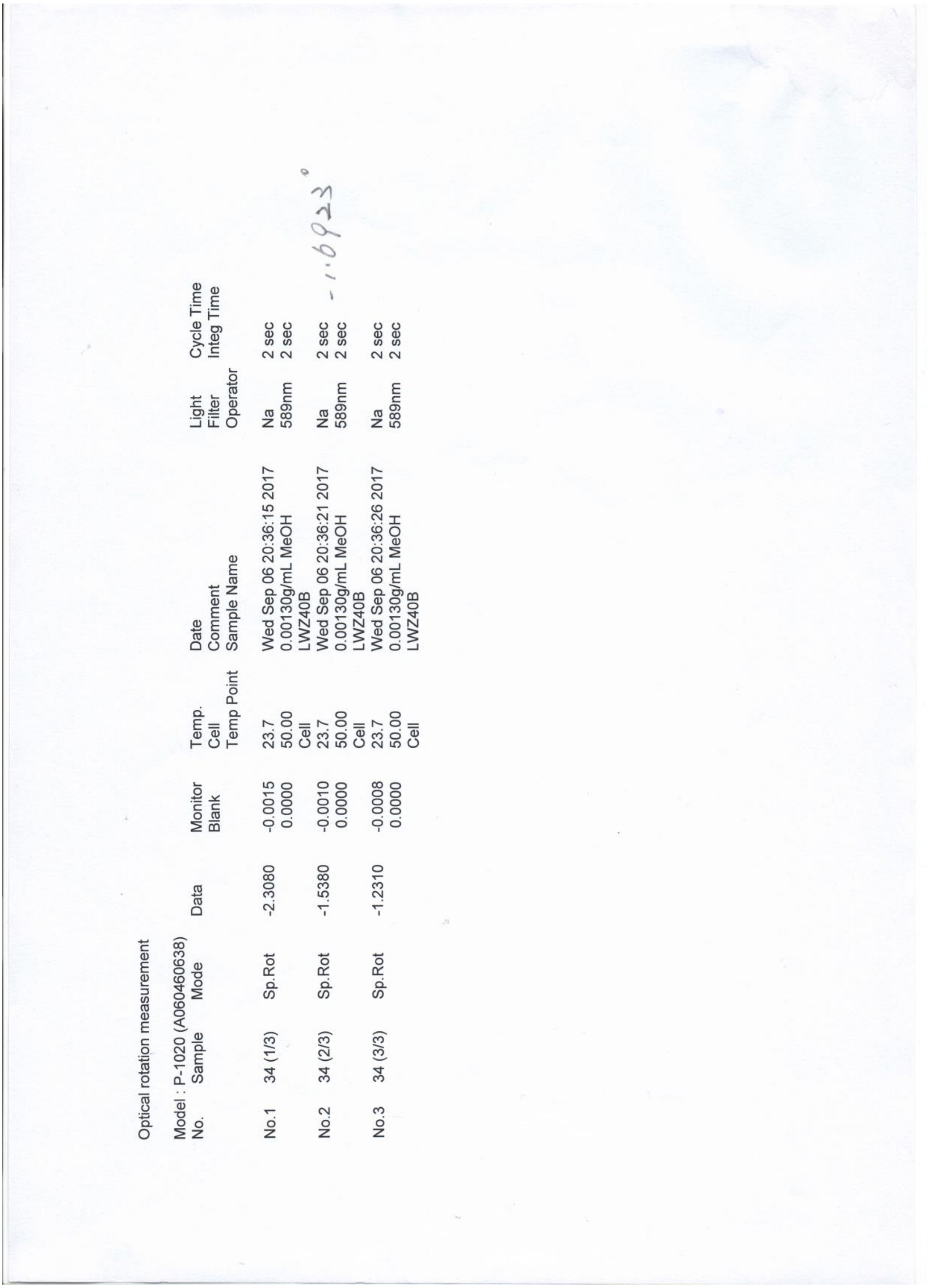
Optical rotation measurement

Model : P-1020 (A060460638)

No.	Sample	Mode	Data	Monitor<br>Blank	Temp.<br>Cell<br>Temp Point	Date<br>Comment<br>Sample Name	Light<br>Filter<br>Operator	Cycle Time<br>Integ Time
No.1	34 (1/3)	Sp.Rot	-2.3080	-0.0015<br>0.0000	23.7<br>50.00<br>Cell	Wed Sep 06 20:36:15 2017<br>0.00130g/mL MeOH<br>LWZ40B	Na<br>589nm	2 sec<br>2 sec
No.2	34 (2/3)	Sp.Rot	-1.5380	-0.0010<br>0.0000	23.7<br>50.00<br>Cell	Wed Sep 06 20:36:21 2017<br>0.00130g/mL MeOH<br>LWZ40B	Na<br>589nm	2 sec<br>2 sec
No.3	34 (3/3)	Sp.Rot	-1.2310	-0.0008<br>0.0000	23.7<br>50.00<br>Cell	Wed Sep 06 20:36:26 2017<br>0.00130g/mL MeOH<br>LWZ40B	Na<br>589nm	2 sec<br>2 sec

-1.6923°

Figure S18. OR spectrum of compound **2**.

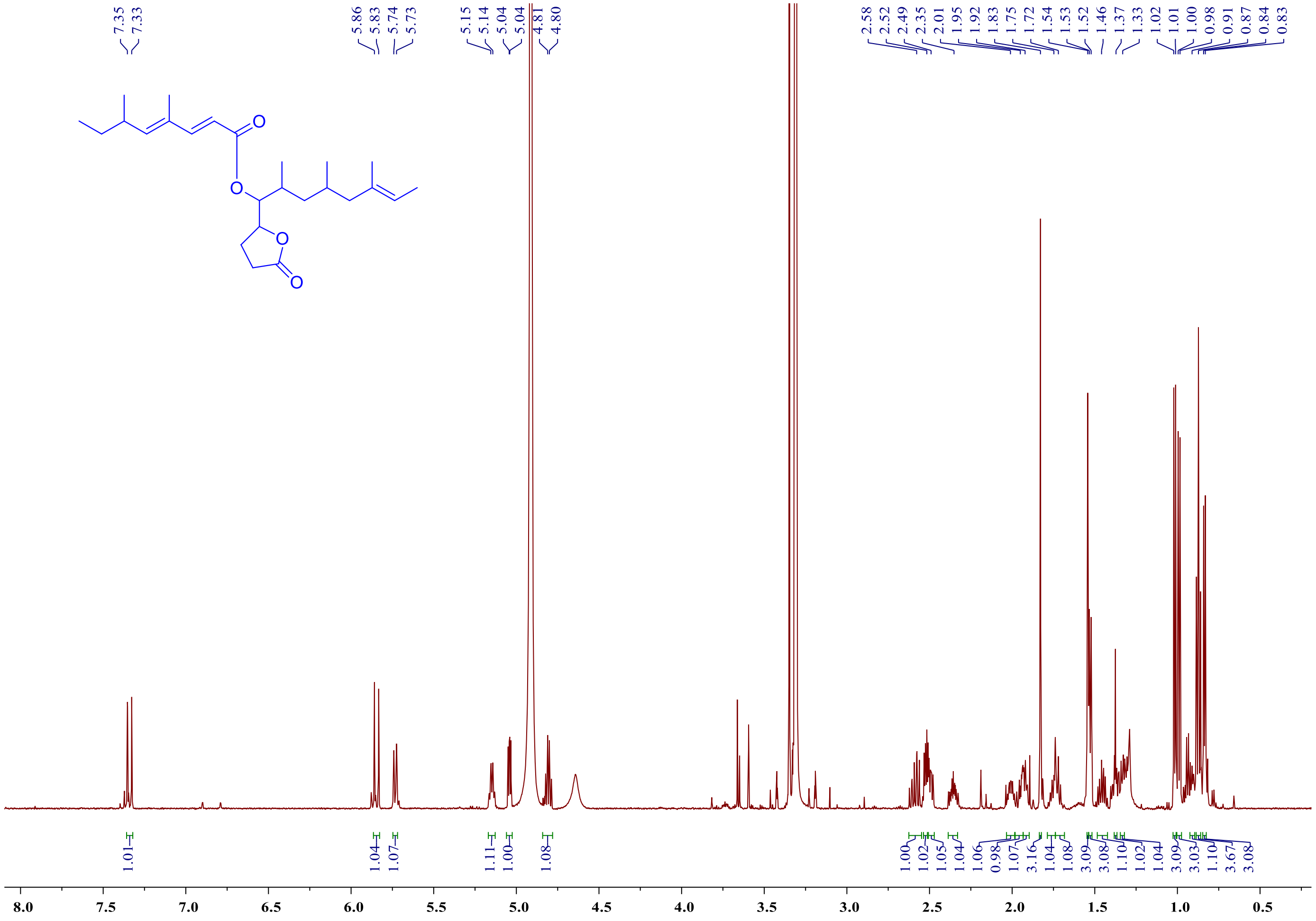

Figure S19. ^{1}H NMR spectrum of compound **3** (CD_3OD).

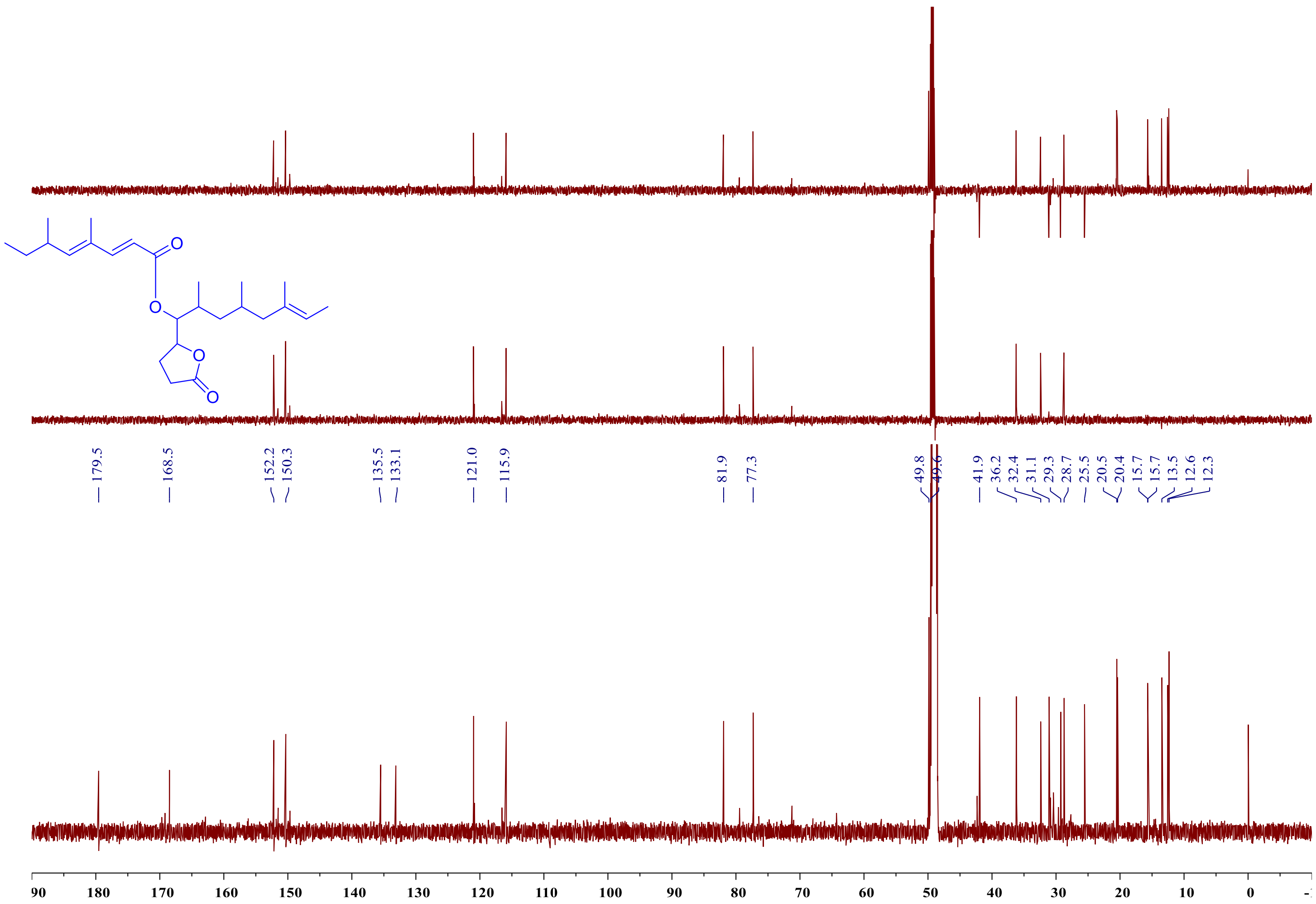

Figure S20. ^{13}C NMR and DEPT spectra of compound **3** (CD_3OD).

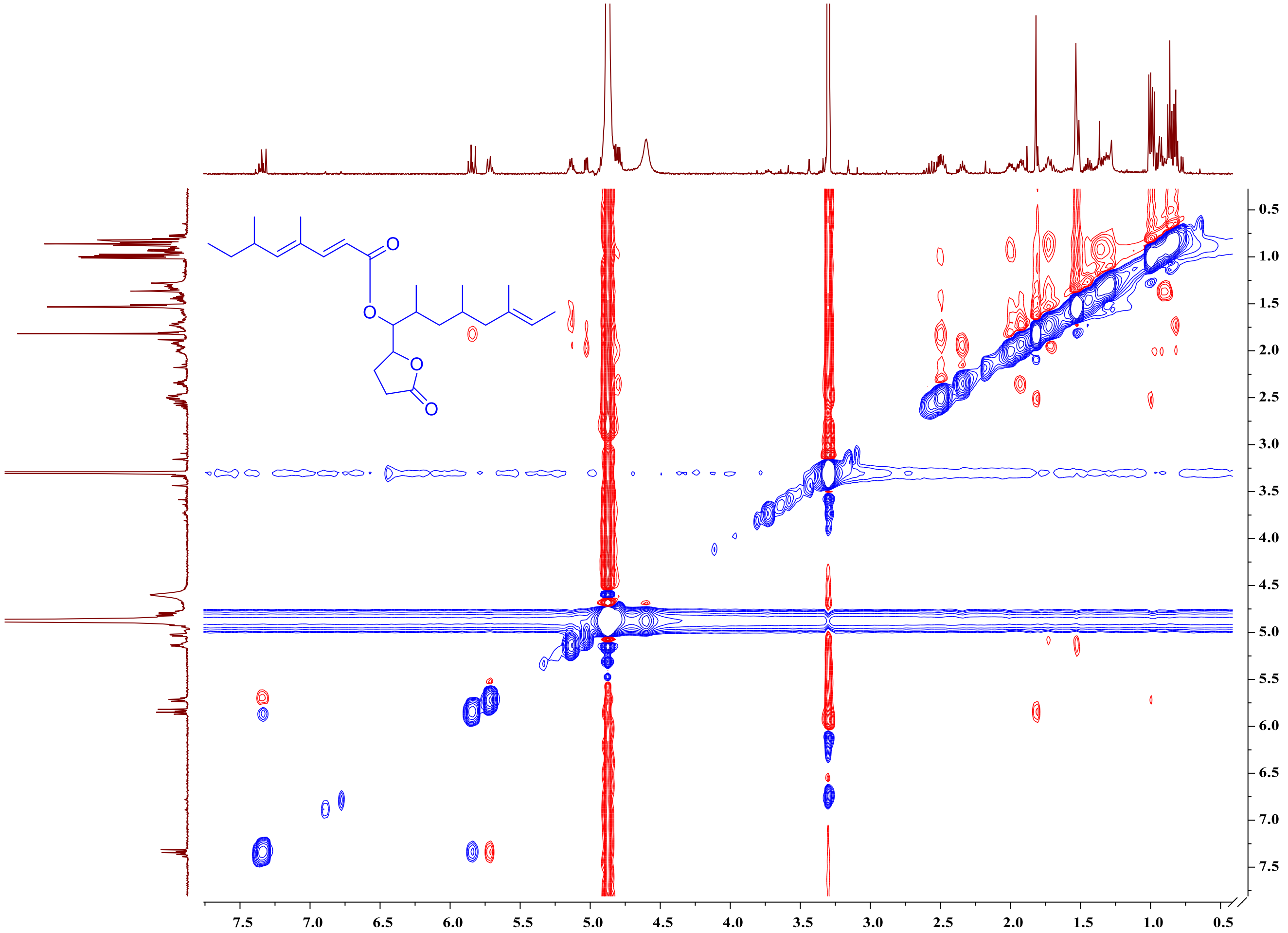

Figure S21. ROESY spectrum of compound **3** (CD_3OD).

Qualitative Analysis Report

**Data Filename**	lwz-12b.d	**Sample Name**	lwz-12b
**Sample Type**	Sample	**Position**	P1-D3
**Instrument Name**	Instrument 1	**User Name**	
**Acq Method**	SIBU.m	**Acquired Time**	7/5/2017 9:31:30 AM
**IRM Calibration Status**	Success	**DA Method**	Default.m
**Comment**			

Sample Group **Info.**
Acquisition SW Version 6200 series TOF/6500 series Q-TOF B.05.01 (B5125.2)

User Spectra

Fragmentor Voltage	Collision Energy	Ionization Mode
135	0	ESI

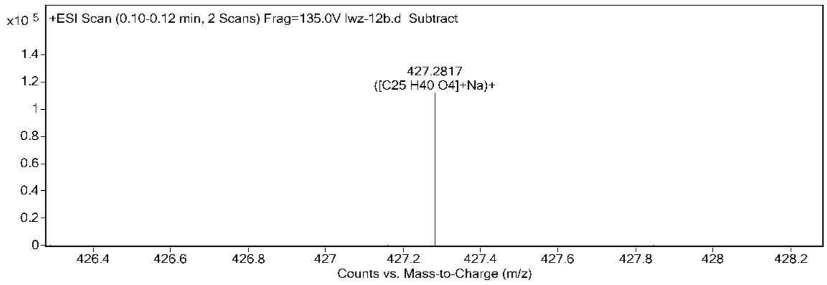

Peak List

m/z	z	Abund	Formula	Ion
422.3265	1	83343.15		
427.2817	1	111310.63	C25 H40 O4	(M+Na)+
443.2557	1	134371.39		
444.2586	1	34756.7		
450.3573	1	71385.59		
459.3067	1	33617.79		
478.3887	1	43320.5		
831.5729	1	53907.6		
847.547	1	55897.48		
863.5992	1	42797.55		

Formula Calculator Element Limits

Element	Min	Max
C	3	60
H	0	120
O	0	30
N	0	5

Formula Calculator Results

Formula	CalculatedMass	CalculatedMz	Mz	Diff. (mDa)	Diff. (ppm)	DBE
C25 H40 O4	404.2927	427.2819	427.2817	0.4	0.9	6.0000

--- End Of Report ---

Figure S22. HRESIMS spectrum of compound **3**.

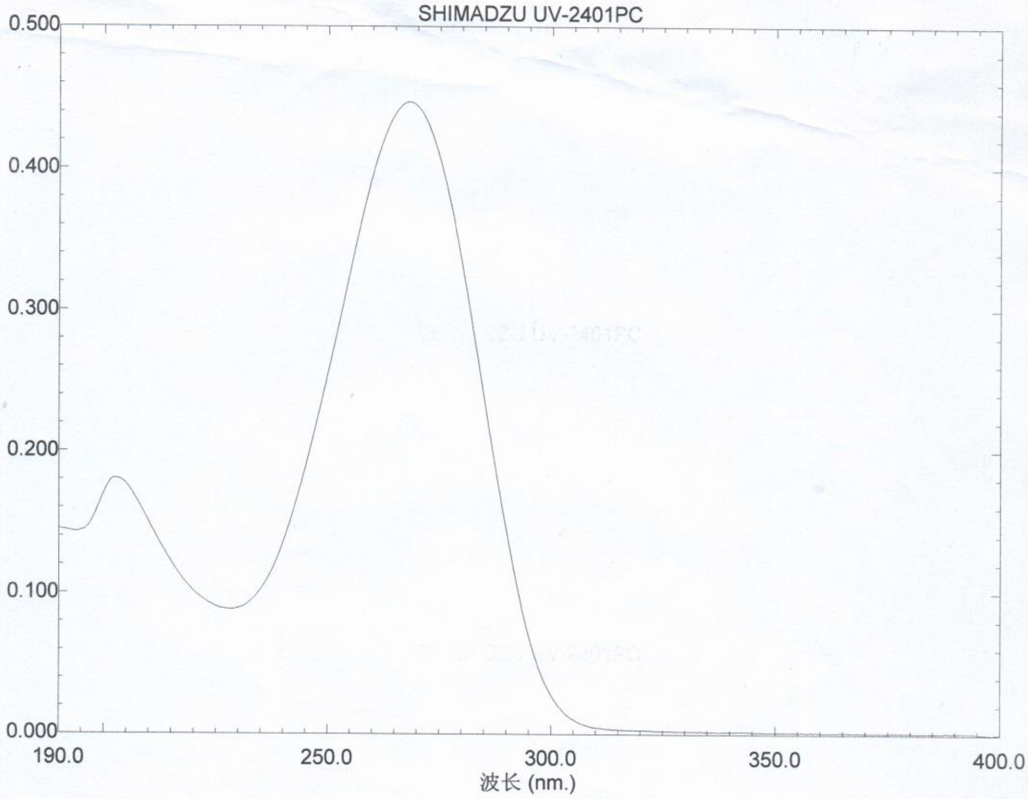

文件名: LWZ12B

LWZ12B———

样品浓度：0.0308毫克/毫升
溶剂：甲醇

创建于: 11:22 17-09-08
数据: 原始

测量模式: Abs.
扫描速度: 中速
狭缝: 5.0
采样间隔: 0.5

否.	波长 (nm.)	Abs.
1	544.50	0.0017
2	268.50	0.4466
3	202.50	0.1805

Figure S23. UV spectrum of compound **3**.

Optical rotation measurement

Model : P-1020 (A060460638)

No.	Sample	Mode	Data	Monitor<br>Blank	Temp.<br>Cell<br>Temp Point	Date<br>Comment<br>Sample Name	Light<br>Filter<br>Operator	Cycle Time<br>Integ Time
No.1	31 (1/3)	Sp.Rot	-3.4290	-0.0012<br>0.0000	23.6<br>50.00<br>Cell	Wed Sep 06 20:28:05 2017<br>0.00070g/mL MeOH<br>LWZ12B	Na<br>589nm	2 sec<br>2 sec
No.2	31 (2/3)	Sp.Rot	-6.5710	-0.0023<br>0.0000	23.7<br>50.00<br>Cell	Wed Sep 06 20:28:11 2017<br>0.00070g/mL MeOH<br>LWZ12B	Na<br>589nm	2 sec<br>2 sec
No.3	31 (3/3)	Sp.Rot	-3.4290	-0.0012<br>0.0000	23.7<br>50.00<br>Cell	Wed Sep 06 20:28:16 2017<br>0.00070g/mL MeOH<br>LWZ12B	Na<br>589nm	2 sec<br>2 sec

-4.4762°

Figure S24. OR spectrum of compound **3**.
